# Comparative transcriptomic insights into iron deficiency response in contrasting rice varieties at the seedling stage reveal distinct response strategies and identify novel candidate genes

**DOI:** 10.3389/fpls.2026.1764898

**Published:** 2026-03-12

**Authors:** Ananya, Mohd Fayaz, Siddharth Panda, Mahender Anumalla, Chandrashekhar B. A., Sanjana B. Sreenivas, Siddaraju R., Nethra N., Annamalai Anandan, Ramanathan Sowdhamini

**Affiliations:** 1 National Centre for Biological Sciences (TIFR), Bengaluru, Karnataka, India; 2 Indian Council of Agricultural Research (ICAR)–Central Rice Research Institute (NRRI), Cuttack, Odisha, India; 3 Rice Breeding Innovation Platform, International Rice Research Institute (IRRI), Los Baños, Laguna, Philippines; 4 University of Agricultural Sciences, GKVK, Bengaluru, Karnataka, India; 5 ICAR–National Institute of Seed Science & Technology, Mau, Uttar Pradesh, India

**Keywords:** DEGs, DSR, hydroponics, iron deficiency, LalatMas, RA23, SPAD

## Abstract

Iron deficiency is a major constraint in rice cultivation, particularly under direct-seeded rice (DSR) systems, where aerobic soil conditions reduce iron availability in plant-accessible forms. In this study, the experiment was initiated with screening of 116 germplasm lines of two weeks old line under hydroponics iron-deficient and sufficient conditions. Based on morphological and SPAD (Soil Plant Analysis Development) values, two contrasting rice genotypes - RA23 (tolerant) and LalatMas (susceptible) were selected and investigated to determine the molecular basis of iron deficiency response through comparative transcriptome analyses. A substantial number of differentially expressed genes (DEGs) were identified in each genotype, revealing distinct transcriptional reprogramming associated with iron acquisition, transport, and homeostasis. Functional classification and enrichment analyses uncovered genotype-specific regulation of pathways related to iron ion transport, general defense and stimulus-response functions, and ADP-binding activity, indicating the involvement of signaling and regulatory proteins. A subset of candidate genes, including both known iron-responsive regulators and previously uncharacterized proteins, was further prioritized based on differential expression patterns, interaction predictions, structural features and expression profiles. Quantitative real-time PCR validation confirmed the expression patterns of selected DEGs, supporting their relevance in iron stress adaptation. Notably, the greater induction of iron transporters in LalatMas likely reflects its stronger compensatory drive to acquire iron relative to RA23. This study provides the first report of leaf-specific transcriptomic signatures associated with iron deficiency in rice and offers a valuable set of candidate genes for functional analysis and breeding of iron-efficient cultivars optimized for DSR and other nutrient-limited agroecosystems.

## 1 Introduction

Iron (Fe) is an essential micronutrient for plant growth and development, playing critical roles in various physiological and biochemical processes such as respiration, photosynthesis, and chlorophyll biosynthesis. As a redox-active element, iron functions as a cofactor in electron transport chains and enzymatic reactions vital for ATP synthesis and the management of reactive oxygen species (ROS) (Kobayashi and Nishizawa, 2012). Within chloroplasts, iron is integral to the formation of iron-sulfur (Fe-S) clusters that are part of the photosystem I complex. A deficiency in this micronutrient leads to impaired chlorophyll biosynthesis, often resulting in interveinal chlorosis in young leaves, a classic symptom of iron deficiency stress (Jeong and Guerinot, 2009; Panda et al., 2024).

Despite its relative abundance in the Earth’s crust, the availability of iron to plants is highly constrained, particularly in aerobic soils where Fe^2+^ (ferrous iron), the plant-available form, is rapidly oxidized to Fe^3+^ (ferric iron), which precipitates as insoluble hydroxides (Marschner, 1995). This limitation is exacerbated in direct-seeded rice (DSR) systems, which are gaining popularity as sustainable alternatives to traditional puddled transplanting due to reduced water usage and labor. However, DSR conditions are characterized by elevated oxygen availability in the root zone, leading to enhanced oxidation of iron, which severely limits its uptake by rice roots (Fageria et al., 2008). Under iron-limited aerobic production systems, plants frequently develop iron-deficiency-induced chlorosis, marked by impaired chlorophyll biosynthesis and reduced photosynthetic capacity, which in turn suppress overall growth and yield potential (Li et al., 2021). The deficiency observed during the early stages of growth adversely affects seedling establishment, complicating efforts to maintain a sufficient population to achieve the desired yield and mitigate weed pressure in Direct Seeded Rice (DSR) systems. Understanding and addressing iron deficiency in rice necessitates a multidisciplinary approach, integrating plant physiology, molecular biology, and genomics. Rice, as a staple crop for over half the global population, is an ideal model for studying iron homeostasis due to its well-characterized genome, rich germplasm resources, and the availability of functional genomics tools. The ability of different rice genotypes to tolerate low-iron conditions varies significantly, with genetic differences in iron uptake, transport, sequestration, and stress response mechanisms playing key roles (Ricachenevsky and Sperotto, 2014; Wang et al., 2020).

Previous studies have identified several key components of the iron acquisition and homeostasis network in rice. Iron uptake at the root surface is mediated largely by transporters such as *OsIRT1* and *OsIRT2*, which facilitate Fe^2+^ absorption under deficiency (Bughio et al., 2002; Ishimaru et al., 2006), and *OsNRAMP1/OsNRAMP5*, which contribute to Fe^2+^ and Mn^2+^ transport (Takahashi et al., 2011; Sasaki et al., 2012). The solubilization and mobilization of Fe^3+^ involve the phytosiderophore pathway, including *OsNAS1/OsNAS2/OsNAS3* for nicotianamine synthesis and *OsDMAS1* for deoxymugineic acid biosynthesis (Inoue et al., 2003; Bashir et al., 2006). Transporters such as *OsTOM1* and *OsYSL15* play essential roles in the secretion of phytosiderophores and the uptake of Fe(III)–DMA complexes (Nozoye et al., 2011; Inoue et al., 2009; Panda et al., 2021), while *OsYSL2* facilitates long-distance transport of Fe–nicotianamine complexes through the phloem (Koike et al., 2004). Internal iron distribution and storage are regulated by vacuolar iron transporters *OsVIT1/OsVIT2* (Zhang et al., 2012) and plastid ferritins *OsFER1/OsFER2* (Stein et al., 2009). Transcriptional regulation of these pathways is primarily governed by bHLH factors such as *OsIRO2*, *OsIRO3*, and *OsbHLH156* (Ogo et al., 2007; Zheng et al., 2010; Zhang et al., 2017), highlighting a coordinated genetic network that supports iron uptake, mobilization, and homeostasis in rice.

This study aimed to elucidate the genetic and transcriptomic basis of iron deficiency tolerance in rice by comparing two contrasting genotypes: RA23, a tolerant line, and LalatMas (LM), a susceptible one. These genotypes were selected based on prior phenotypic screening under low-iron conditions, in which RA23 consistently showed superior growth, reduced chlorosis, and higher tissue iron concentrations compared with LM (Panda et al., 2024). Despite extensive advances in understanding root-mediated iron uptake, leaf-specific molecular responses underlying visible foliar symptoms remain less explored. To address this gap, comprehensive transcriptomic investigation of iron deficiency responses in rice leaves was conducted, enabling new insights into systemic signaling and stress adaptation beyond the root level.

Transcriptome profiling provides a powerful approach to capture genome-wide expression changes under nutrient stress and to uncover both shared and genotype-specific responses. Functional categorization and enrichment of differentially expressed genes (DEGs) commonly highlight pathways associated with ion transport, redox regulation, metal homeostasis, and stress signaling, all of which play central roles in iron deficiency adaptation. Previous studies have demonstrated the involvement of transporters, chelators, reductases, and several transcription factor families—including bHLH, MYB, and WRKY—in regulating iron uptake and mobilization in rice (Ogo et al., 2007; Zhang et al., 2017; Ricachenevsky and Sperotto, 2014). These components form part of a broader regulatory framework that coordinates iron acquisition and internal redistribution under limiting conditions. Recent studies have highlighted the pivotal role of transcription factor–mediated regulation in iron deficiency responses. Notably, WRKY transcription factors have been shown to exhibit differential regulation and functional interactions under iron deficiency conditions. A recent study in hexaploid wheat demonstrated that WRKY transcription factor homeologs are differentially regulated and interact in a coordinated manner to modulate iron deficiency responses, integrating transcriptional control with physiological and biochemical adaptations (Saini et al., 2025). These findings underscore the conserved and multilayered regulatory roles of WRKY family members in iron homeostasis and provide a comparative framework for understanding genotype-specific regulatory strategies in rice under iron deficiency.

In addition to well-characterized regulators, transcriptome analyses frequently reveal numerous hypothetical or uncharacterized genes whose functions remain unknown. Such genes represent an underexplored layer of the iron deficiency response and may contribute to genotype-specific tolerance mechanisms. Integrating differential expression patterns with functional prediction tools, structural annotations, and interaction information can assist in prioritizing these candidates for downstream studies. In hexaploid wheat, integrated transcriptome and biochemical analyses have been used to dissect contrasting genotypic responses to iron deficiency. Saini et al. (2025) performed transcriptomic profiling alongside biochemical characterization in wheat genotypes differing in iron deficiency tolerance and identified key transcriptional networks and metabolic adjustments that underlie differential tolerance. This cross-species evidence supports the notion of conserved molecular responses to iron deficiency and strengthens the comparative framework for interpreting genotype-specific transcriptomic patterns in rice.

Overall, a comprehensive investigation that combines comparative transcriptomics with functional annotation and candidate gene prioritization provides an opportunity to improve our understanding of iron deficiency adaptation in rice. Insights gained from such analyses are particularly valuable for direct-seeded rice (DSR) systems, where iron availability is inherently limited. By dissecting the contrasting responses of RA23 and LM, this study contributes to the identification of key pathways and potential regulators that may support the development of iron-efficient rice varieties suited to iron-deficient agroecosystems. Although several components of the iron homeostasis network have been characterized, uncharacterized DEGs emerging from transcriptomic studies continue to offer promising leads for discovering previously unknown regulators of iron stress tolerance.

## 2 Methods

The present study was conducted in two distinct phases to identify and validate genes associated with iron (Fe) deficiency tolerance in rice. In Phase I, 116 rice genotypes were screened (**Supplementary File 1**) under hydroponic conditions with iron deficiency (0S). Subsequently, nine genotypes were selected and assessed under both iron-sufficient (100S) and iron-deficient (0S) conditions in hydroponics to analyze the effects of genotype and their interactions in the presence or absence of iron. Following this, the selected lines underwent transcriptomic profiling to identify differentially expressed genes (DEGs). In Phase II, an independent hydroponic experiment was established under identical conditions to validate the expression of selected candidate genes by quantitative real-time PCR (qRT-PCR). This two-phase design ensured both discovery and independent validation of Fe deficiency–responsive genes.

### 2.1 Plant material, hydroponic setup and transcriptomic profiling

#### 2.1.1 Phase I

##### 2.1.1.1 Experimental design, selection of genotypes, interaction effect, hydroponic pH control and experimental rationale

Seeds of both the genotypes were surface-sterilized using 70% ethanol (1 min) followed by 4% sodium hypochlorite (15 min) and rinsed thoroughly with sterile distilled water. Sterilized seeds were germinated on moist filter paper in petri dishes and maintained in the dark for 3 days at 28 °C. Uniform seedlings were transferred on to the styrofoam placed into plastic containers containing modified Yoshida’s nutrient solution (**Supplementary Table 1**) (Yoshida et al., 1976) for hydroponic growth without iron. To simulate iron deficiency, FeNaEDTA was excluded from the nutrient medium, while the concentrations of macronutrients and other micronutrients remained unchanged. In our hydroponic design, the pH of the nutrient solution was intentionally controlled as part of the treatment protocol: for the 0% iron treatment, the solution pH was maintained at or above 5.5 to ensure minimal bioavailable iron, effectively preventing its uptake (Panda et al., 2024). This pH manipulation is based on the principle that iron solubility decreases at near-neutral pH levels due to precipitation as ferric hydroxides (Römheld and Marschner, 1986; Kobayashi and Nishizawa, 2012). In contrast, for the 100% iron treatments, the solution pH was kept below 4.5 to maximize iron solubility and promote efficient iron uptake. It is important to note that these pH setpoints serve as methodological controls and should be interpreted as treatment parameters (i.e., manipulated solution chemistry), rather than indicators of root-mediated rhizosphere acidification. The pH table (**Supplementary Table 3**) therefore, documents maintenance of the intended hydroponic environments across sampling days (5–18 DAS), validating that the physiological responses reflect the imposed Fe availability regimes. Plants were grown in a greenhouse at CRRI (National Rice Research Institute, Cuttack) under a 16/8 h light/dark cycle with 28 ± 1 °C temperature and 60–70% relative humidity. Nutrient solutions were replaced every six days to maintain consistent ion availability and to minimize microbial contamination.

##### 2.1.1.2 Phenotyping the test material

The iron-deficient (0% iron) treatments were replicated twice and the following morphological traits, including shoot length (cm), maximum root length (cm), number of leaves, and chlorophyll index (SPAD reading of the second leaf), fresh and dry biomass/weight of the leaves were recorded on the 15^th^ day. In highly susceptible plants, a third leaf often emerged, but by the 15th day the first leaf had completely dried. In such cases, the SPAD reading was taken from the third leaf, while the leaf number was still noted as two. For biomass estimation, shoot and root samples were dried in a forced-air oven at 60 °C for 5–6 days until fully dehydrated and expressed in milligrams. To assess how iron deficiency affected root traits, root images were scanned using WinRHIZO Pro 2013e (LA 2400, Regent Instruments Inc.) and traits such as root average diameter (mm), root volume (cm3), total root length (cm), projected root area (cm2), total root surface area (cm2), number of root tips, forks and crossings were recorded. All the 116 genotypes were subjected to PCA analysis, nine better and poor performers were selected, and highly contributing traits under Fe-deficient conditions were studied again under Fe deficient and sufficient conditions to understand the role Fe in trait expression and interaction effect.

###### 2.1.1.2.1 Statistical analysis

The variability in traits related to shoot and root traits in 0% iron under hydroponics was described using a PCA. PCA analysis was carried out using the FactoMine R package (Lê et al., 2008) in R 3.6.4 [R Core Team, 2017 (Team, 2017)]. Euclidean distance between two genotypes was estimated by analyzing PCA in the multivariate space and identification of genotypes having Fe deficiency tolerance under hydroponic conditions. To understand the interaction effect between genotype and concentration, R package FCRD 2 Factor was used (Manivannan, 2014) in R 3.6.4.

##### 2.1.1.3 Transcriptome generation

From the concentration (C) x genotype (G) interaction studies, two contrasting rice genotypes RA23 (tolerant) and LM (susceptible) were selected to investigate transcriptomic responses to Fe deficiency. Similar to phase 1, section 1, the hydroponics setup was executed for Fe-deficient (0% iron) and Fe-sufficient (100% iron) conditions to have samples for transcriptome profiling. Plants were grown in a greenhouse at CRRI, under controlled conditions (28 ± 1 °C, 16/8 h light/dark, 60–70% RH) and nutrient solutions were refreshed every six days, and pH was monitored every alternate day to ensure treatment consistency. On the 15th day, shoot tissues were sampled, surface sterilized in 70% ethanol, washed and they were immediately frozen in liquid nitrogen and stored at –80 °C for RNA isolation and transcriptome sequencing. Sampling strategy included collection of leaf tissues of 15 days old seedlings to capture temporal gene expression dynamics associated with early sensing, signal transduction, and eventual physiological adaptation to iron deficiency. Leaf samples were used for transcriptomic analysis.

### 2.1.2 Phase II: validation experiment

Following transcriptome analysis, a second hydroponic experiment was conducted using the same genotypes (RA23 and LM) and identical nutrient conditions described above. This phase was designed to provide independent biological replicates for validating the expression of selected DEGs by qRT-PCR. All hydroponic parameters—nutrient composition, iron concentrations, pH range, photoperiod, and temperature—were maintained at ICAR-NISST, Bangalore station and NCBS, Bangalore identical to Phase I to ensure direct comparability between transcriptomic and experimental datasets. Leaf tissues were collected at 10^th^, 15^th^ and 20^th^ days post-treatment, corresponding to the peak iron deficiency response stage identified from Phase I expression data. These samples were used for RNA extraction, cDNA synthesis, and quantitative expression validation.

### 2.2 Phenotypic and physiological assessment

Phenotypic monitoring of plants under different iron regimes was conducted to establish a link between observable symptoms and molecular responses. Plants were visually assessed for typical iron deficiency symptoms such as interveinal chlorosis in young leaves, reduced root growth, reduced leaf number, and overall biomass reduction. For this morphological analysis, readings were collected from five biological replicates at all three time points under both iron-deficient and iron-sufficient conditions, and standard deviations were calculated for each condition and time point. To quantitatively assess iron deficiency effects, SPAD (Soil Plant Analysis Development) meter readings were taken from the second and third fully expanded leaves. SPAD values are widely used to estimate chlorophyll content and serve as a proxy for iron nutritional status in rice (Zhao et al., 2022; Panda et al., 2024). Additionally, pH of the nutrient solution was recorded every two days to monitor potential pH drift due to root exudation and henceforth maintained (**Supplementary Table 2**), as Fe solubility is highly pH-sensitive.

### 2.3 Transcriptome analysis, RNA extraction, cDNA library preparation

#### 2.3.1 Transcriptome analysis - RNA-seq quality control and mapping

Raw RNA-seq reads generated from shoot tissues of LM and RA23 were subjected to quality control using fastp (Chen et al., 2018), which performed adapter trimming, filtering of low-quality bases, and removal of artifacts. Quality parameters such as Phred score distribution, GC content, and duplication levels were compiled using MultiQC. High-quality reads were then aligned to the *Oryza sativa* reference genome with HISAT2 (v2.1.0) (Kim et al., 2015) under default settings. Gene-level read counts were obtained using featureCounts, which generated assigned and unassigned read fractions for each sample. Differential expression analysis was performed using DESeq2, including normalization of raw counts, estimation of dispersion, and statistical testing using the Wald method. Genes were considered differentially expressed at |log2FC| > 1 and padj < 0.05. Further, to visualize dataset overlap and sample-level variation, Venn diagrams were generated using jvenn (Bardou et al., 2014), volcano plots were generated using Matplotlib (Hunter, 2007) and Seaborn (Waskom, 2021) libraries in Python. Principal component analysis (PCA) was also performed to assess sample clustering and experimental reproducibility (Jolliffe and Cadima, 2016) to highlight significant outliers and guide downstream candidate prioritization.

#### 2.3.2 RNA extraction and cDNA library preparation

Following phenotypic and physiological evaluations, leaf tissues from both genotypes (RA23 and LM) were harvested for RNA extraction. To minimize circadian bias in gene expression, samples were collected at the same time in the morning, as gene expression in plants is known to follow a circadian rhythm that can significantly alter transcript abundance (Covington et al., 2008; Michael et al., 2008). Immediately after harvesting, leaf samples were snap-frozen in liquid nitrogen to prevent RNA degradation by rapidly inactivating RNases (Rio et al., 2010) and stored at −80 °C to preserve RNA integrity until extraction (Sambrook and Russell, 2001). Leaf tissues (50–100 mg) were collected on the 15th day (in phase I) and 10, 15, and 20 days after germination (in phase II). Total RNA extraction was performed using Trizol reagent (Invitrogen), and the samples were stored at −80 °C for subsequent analyses. The quality and quantity of the extracted RNA were assessed using agarose gel electrophoresis and a Nanodrop spectrophotometer (NanoPhotometer NP80). To eliminate genomic DNA contamination, the RNA samples were treated with RNase-free DNase I (Biofoundry). First-strand cDNA synthesis was conducted using 2–3 µg of RNA with the RevertAid H Minus First Strand cDNA Synthesis Kit and oligo(dT) primers (Thermo, USA).

### 2.4 Candidate gene prioritization and novel candidate identification

#### 2.4.1 Candidate gene prioritization

To identify robust iron-responsive candidates, DEGs were filtered using stringent thresholds for statistical significance (FDR-corrected p-value) and biological relevance (high absolute log_2_ fold change). Volcano plots were then examined to capture outliers showing strong induction under iron deficiency, as these represent the most responsive genes in each comparison. Annotated gene identities were retrieved from RAP-DB and related rice annotation resources, allowing us to distinguish well-characterized iron-associated genes from those lacking functional annotation. Notably, several highly upregulated outliers were listed in RAP-DB as hypothetical proteins or DUF-containing proteins, indicating potentially novel components of the iron-deficiency response. Representative genes from this refined set were selected for RT-qPCR validation, and particular emphasis was placed on these uncharacterized upregulated candidates to enable further functional characterization and uncover their potential roles in iron homeostasis. Uncharacterized genes were further examined using STRINGdb (PPI analysis), PSI-BLAST, and HHPred to detect distant homologs or structural analogues.

##### 2.4.1.1 Protein–protein interaction network analysis

To understand the functional connectivity among differentially expressed genes (DEGs) and to identify key regulatory hubs involved in iron deficiency responses, protein–protein interaction (PPI) networks were constructed using STRING v12.5 (Szklarczyk et al., 2023). Individual DEGs from all major pairwise comparisons (0S_0T, 0S_100S and 0T_100T) were submitted to the STRING database one by one, using *Oryza sativa* (IRGSP-1.0) as the reference organism. To ensure biological reliability, only edges supported by experimental evidence, curated databases, or text-mining with co-expression validation were retained. Protein–protein interaction (PPI) networks were visualized and analyzed in Cytoscape (Shannon et al., 2003), an open-source platform for biological network analysis. Network topological parameters, including node degree (number of connections), betweenness centrality (measure of information flow control), and clustering coefficient (extent of local interconnectivity), were calculated using the NetworkAnalyzer plugin (Assenov et al., 2008). Nodes representing DEGs with high centrality or hub-like behavior were prioritized for further functional characterization, as they may serve as regulatory bottlenecks or signaling integrators in the iron deficiency response pathway.

#### 2.4.2 Novel gene identification - distant homology and structure-based annotation

To functionally annotate novel hypothetical DEGs, we applied complementary sequence- and structure-based homology prediction approaches. PSI-BLAST (three iterations) was used to detect remote sequence homologs by building PSSMs capable of identifying evolutionarily conserved matches that standard BLASTp cannot capture. In parallel, HHPred (Söding et al., 2005) was employed to predict structural homology through HMM–HMM comparisons against curated databases such as PDB, Pfam-A, SCOP and COG. Confident matches were defined using HHPred probability ≥ 75% and E-values ≤ 1e−5. Integrating PSI-BLAST and HHPred outputs—along with domain information and predicted structural models—allowed functional inference for otherwise uncharacterized DEGs. This combined strategy improved interpretability of lineage-specific or DUF-containing proteins implicated in the iron-deficiency response. One novel candidate from each dataset (0S vs 100S and 0S vs 0T) was subsequently selected for RT-qPCR validation.

### 2.5 Functional annotation and gene ontology enrichment analysis

To gain a systems-level understanding of the molecular mechanisms underlying iron deficiency responses, comprehensive functional annotation and pathway enrichment analysis was performed on the differentially expressed genes (DEGs). GO term enrichment was conducted using GOATOOLS, a Python-based toolkit for Gene Ontology analysis (Klopfenstein et al., 2018). Over-representation analysis was performed using g:Profiler (version e113_eg59_p19_f6a03c19) with the g:SCS multiple testing correction method and a significance threshold of 0.05 (Kolberg et al., 2023). Importantly, the full set of 34,208 expressed genes detected across all samples was used as the background gene universe, ensuring that enrichment was evaluated relative to all genes actively expressed under our experimental conditions rather than the entire rice genome. Annotation was supplemented using RAPDB, Ensembl BioMart (Kinsella et al., 2011), and a curated set of iron-related GO terms from published literature was mapped onto the DEGs to highlight biologically relevant pathways. GO terms were categorized into Biological Processes (BP), Molecular Functions (MF), and Cellular Components (CC). Fisher’s exact test with FDR correction was applied and GO terms with adjusted p < 0.05 were retained.

### 2.6 Quantitative real-time PCR validation and expression analysis

#### 2.6.1 Quantitative real-time PCR validation

To validate the reliability of RNA-seq–based differential expression results, quantitative real-time PCR (qRT-PCR) was performed on a representative panel of 18 candidate genes. This panel included both well-characterized Fe-responsive marker genes and novel uncharacterized genes, such as those containing domains of unknown function (DUFs) or hypothetical annotations. qRT-PCR offers high sensitivity and specificity for confirming transcript abundance across independent biological replicates (Bustin et al., 2009). By selecting genes from both high-confidence known regulators and newly emerging candidates, this validation step aimed to confirm the robustness of RNA-seq–derived expression trends and support the discovery of potentially novel components involved in the iron deficiency stress response in rice.

#### 2.6.2 qRT-PCR based expression analysis

qRT-PCR–based expression analysis was performed on a Bio-Rad CFX96 real-time PCR system. Each 10 µL reaction consisted of 3.4 µL nuclease-free water, 5 µL PlantAce QPCR Elite Dye Master Mix (2X Premix, Biofoundry) with 0.2 µL ROX reference dye (50X), 1 µL of normalized cDNA, and 0.2 µL each of gene-specific forward and reverse primers. The thermal cycling conditions included an initial denaturation at 95 °C for 10 min, followed by 40 cycles of 95 °C for 15 s and extension at 60 °C for 1 min, followed by a melt curve analysis. After performing qRT-PCR, the Ct values obtained for the target gene and the internal control gene (OsActin) were recorded to assess their expression levels. These Ct values were then used to calculate the relative fold change in gene expression using the 2-ΔΔCt method outlined by Livak and Schmittgen (2001). Information on the primers used for the qRT-PCR analysis is listed in **Supplementary File 2**.

## 3 Results

### 3.1 Principal component analysis of the 116 test genotypes used for preliminary screening

**Figure 1:**
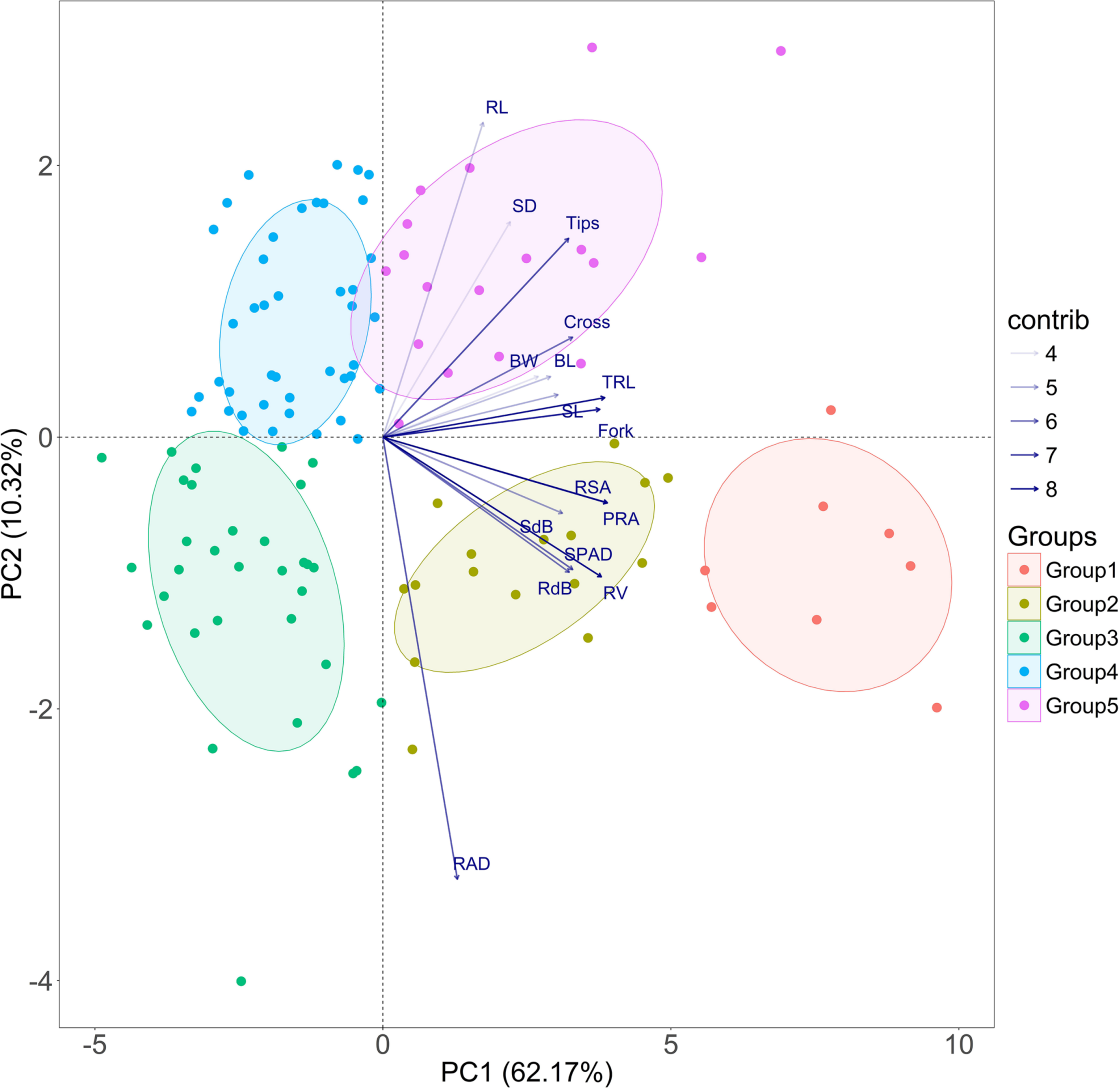
Principal component analysis (PCA). It was conducted on shoot and roots traits several traits, including shoot length (SL), maximum root length (RL), stem diameter (SD), blade length (BL), blade width (BW), shoot biomass (SdB), root biomass (RdB), chlorophyll index (SPAD), root average diameter (RAD), root volume (RV), total root length (TRL), projected root area (PRA), total root surface area (RSA), number of root tips, forks, and crossings (Cross) under hydroponic conditions. The two principal component axes accounted for 73% of the total variance in the dataset. The transparency of each vector indicates its contribution to the variance, with lighter tones representing contributions of 4% and darker tones indicating contributions of 8%. The direction and length of each vector reflect the traits’ contributions to the first two components of the PCA. Genotypes were categorized into five groups based on their expression patterns of shoot and root traits measured under hydroponic conditions. Group 1 (solid red tone) and Group 2 (solid algae tone) consist of genotypes that exhibit excellent and good tolerance to iron deficiency, while Group 3 (solid green tone) and Group 4 (solid cyan tone) are susceptible under 0% iron. Group 5 (solid purple tone) displays moderate performance under 0% iron at 14 days.

Principal component analysis of the trait dataset (116 genotypes) showed that the first principal component (PC1) had an eigenvalue of 9.947 and explained 62.17 percent of the total variation, which clearly indicates that most of the variability among the traits was captured along this single dominant axis. PC2 accounted for a further 10.32 percent, and PC3 contributed 6.38 percent, bringing the cumulative variation explained by the first three components to 78.87 percent (**Figure 1**). PC1 was mainly influenced by traits related to overall plant vigor and root system development, with high contributions from projected root area, root surface area, total root length, root volume and number of forks. Shoot traits such as SPAD, shoot dry biomass and shoot length also loaded strongly on this component, suggesting that PC1 largely captured general growth intensity. PC2 was driven primarily by root length, stem diameter and root tips, indicating that this axis represented differences in stem thickness, root tips and root length suggesting variation associated with finer branching and elongation behavior. PC3 showed high loadings for leaf blade length and shoot length, reflecting variation associated with aerial traits such as shoot elongation and leaf expansion.

#### 3.1.1 Anova and interaction studies

Analysis of Variance was carried out for the top five contributing traits identified through PCA, namely projected root area, root surface area, total root length, second leaf width and SPAD to understand the interaction between Fe concentration and genotype. Factor A (Fe concentration; 0 and 100 percent) had a highly significant effect (P < 0.001) on all five traits. Replication effects were non-significant for all traits except SPAD, which showed a small but significant effect (P = 0.039). Factor B (genotypes) did not significantly influence projected root area, root surface area or total root length, but had a strong and highly significant effect on second leaf width and SPAD. The A × B interaction was non-significant for the root traits, indicating stable responses of these parameters across genotypes. However, significant interaction effects were observed for second leaf width and SPAD, suggesting that these shoot traits responded differently across genotypes under varying Fe concentrations. Error variances were low across all traits, reflecting good experimental precision.

### 3.2 Phenotyping RA23 and Lalat MAS

Phenotypic evaluation under contrasting iron regimes revealed clear varietal differences between RA23 (tolerant) and LM (susceptible) across all measured traits. A combination of visual assessments, dimensional measurements, and biomass quantification provided a comprehensive understanding of the morphological response to iron deficiency.

#### 3.2.1 Root and shoot length responses under varying iron regimes

**Figure 2:**
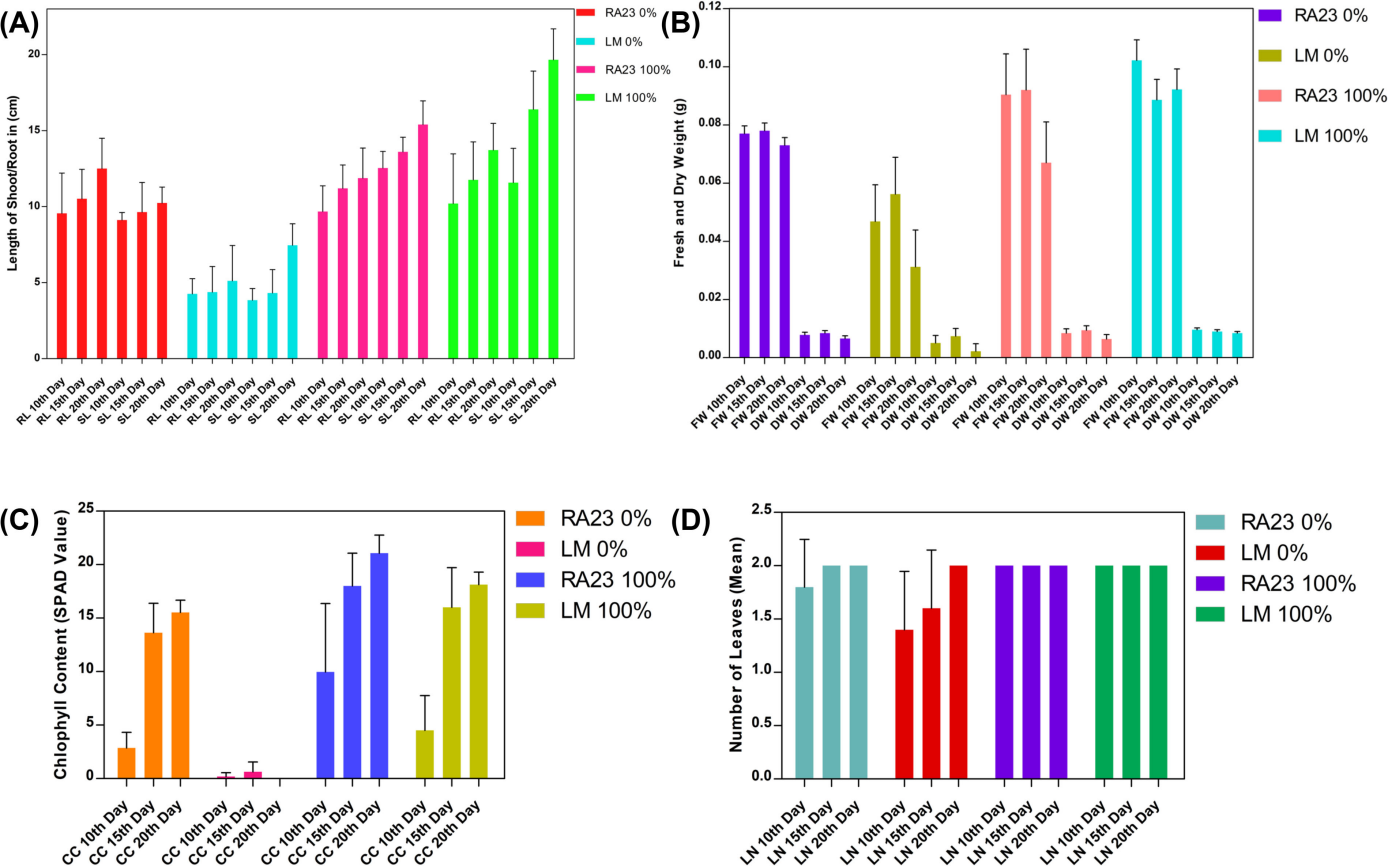
Effect of iron deficiency on morphological traits in RA23 and LalatMAS seedlings. **(A)** Shoot length (SL) and root length (RL) (n = 5). **(B)** Fresh weight (FW) and dry weight (DW) (pooled sample of 5 seedlings per treatment). **(C)** Chlorophyll content (CC; SPAD values) (n = 3). **(D)** Leaf number (LN) (n = 5) under control and iron-deficient conditions at different time points, where n represents the number of biological replicates/sample size. Values represent means derived from the indicated number of biological replicates or pooled samples. Individual replicate values and details of pooling are provided in **Supplementary File 5**.

Root length (RL) and shoot length (SL) measurements further highlighted the contrasting growth response between the two varieties (**Figure 2A**). Under 0% iron, RA23 seedlings retained comparatively better growth, with RL values of 9–12 cm and SL values of 9–10 cm across the time course. In contrast, LM exhibited severe growth inhibition, with RL restricted to 4–7 cm and SL to 4–8 cm, confirming heightened sensitivity to iron deficiency. Under 100% iron, both varieties showed marked improvement in growth, but RA23 again performed better. RA23 roots elongated progressively from ~9 cm at Day 10 to ~15.5 cm at Day 20, while shoots grew to ~14–15 cm by Day 20. LM also showed enhanced growth under adequate iron, with RL reaching ~12 cm and SL ~19–20 cm by Day 20, though still lagging behind RA23. These observations underscore the differential growth resilience of the two genotypes, with RA23 exhibiting superior maintenance of both root and shoot elongation under iron-limited conditions.

#### 3.2.2 Fresh and dry biomass accumulation under iron-deficient and iron-sufficient conditions

Biomass measurements revealed substantial variation in both fresh weight (FW) and dry weight (DW) between iron-deficient and iron-sufficient plants, with strong genotype-specific differences (**Figure 2B**). Under 0% iron, RA23 maintained relatively higher FW (0.075–0.08 g) across all time points compared to LM, which showed markedly reduced FW (0.045–0.06 g). Dry weight followed a similar pattern: RA23 retained DW values of 0.006–0.008 g, whereas LM’s DW dropped sharply to 0.002–0.004 g, indicating greater biomass loss in the susceptible genotype. In contrast, plants grown under 100% iron exhibited substantial improvements in biomass. RA23 showed a pronounced increase in FW (0.085–0.095 g) and DW (0.006–0.009 g), reflecting efficient biomass accumulation under sufficient iron. LM also demonstrated improved growth under 100% iron, maintaining FW values of 0.09–0.105 g and DW of 0.005–0.007 g, though still slightly lower than RA23. Biomass accumulation increased progressively from Day 10 to Day 20 in both genotypes under sufficient iron.

**Figure 3:**
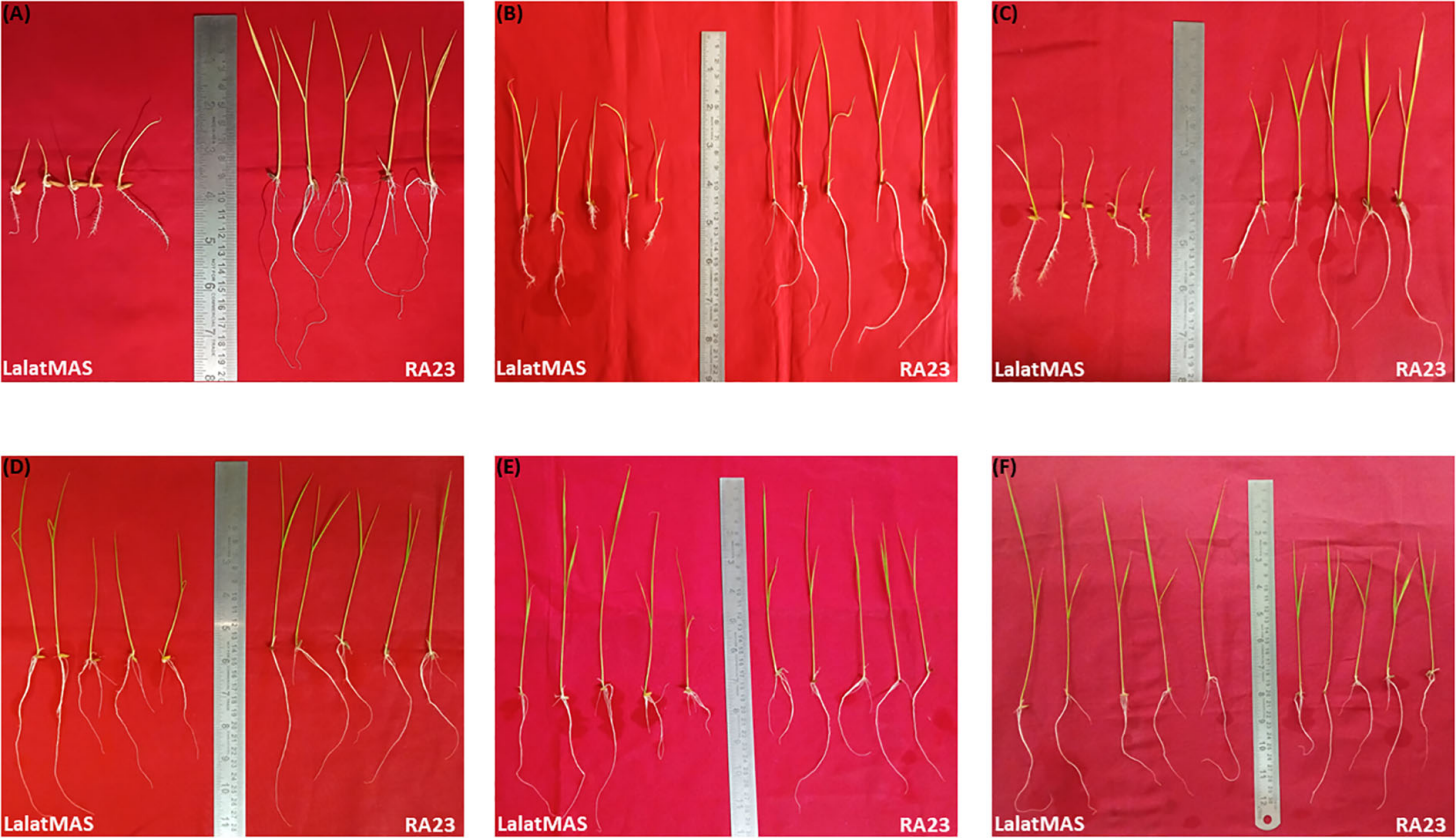
Phenotypic response of Lalat MAS and RA23 at 10^th^, 15^th^, and 16^th^ days after sowing in response to 0% iron (iron deficient) and 100% (iron sufficient) hydroponic medium **(A)** 10^th^ day in 0% iron, **(B)** 15^th^ day in 0% iron, **(C)** 20^th^ day in 0% iron, **(D)** 10^th^ day in 100% iron, **(E)** 15^th^ day in 100% iron, **(F)** 20^th^ day in 100% iron.

Iron deficiency progressively heightened the differences in growth between LM and RA23 across all time points. Under 0% iron, LM consistently showed severe stress symptoms, including short shoots, visible chlorosis, and poorly developed, fragile roots from 10^th^ day through 15^th^ and 20^th^ day (**Figures 3A–C**). In contrast, RA23 maintained better growth, with longer shoots, healthier leaves, and more sustained root elongation, demonstrating greater tolerance to iron deprivation. Under 100% iron, both genotypes exhibited improved growth; however, RA23 continued to show stronger early biomass accumulation and a more robust root system. By Day 20, LM produced taller shoots under sufficient iron, while RA23 allocated more growth to thicker, longer roots. These observations highlight distinct adaptive strategies, with RA23 maintaining superior root development that supports better performance under nutrient stress. Overall, these results confirm that iron deficiency significantly suppresses both fresh and dry biomass in rice, with LM more severely affected, whereas RA23 exhibits comparatively better biomass retention and recovery.

#### 3.2.3 Chlorophyll content (SPAD values) under iron-deficient and iron-sufficient conditions

Chlorophyll content, measured via SPAD readings, showed a clear and consistent separation between iron-deficient (0%) and iron-sufficient (100%) treatments across both varieties (**Figure 2C**). Under 0% iron, LM (LM) exhibited a drastic decline in SPAD values at all three time points, reflecting severe chlorosis while RA23 substantially survived. RA23 displayed apparently higher SPAD values (15 units) compared to LM confirming its better tolerance to iron deficiency induced pigment loss (Chlorosis). In contrast, plants grown under 100% iron maintained substantially higher chlorophyll levels. RA23 showed the highest SPAD values, reaching ~20–25 units by Day 20, whereas LM maintained moderately high values (~16–18 units). Across both varieties, chlorophyll content increased progressively from Day 10 to Day 20, with RA23 showing the most pronounced recovery under sufficient iron. Overall, these results demonstrate that RA23 maintains chlorophyll biosynthesis more effectively than LM under iron stress.

#### 3.2.4 Leaf number variation during the experimental period

Leaf number (LN) remained relatively stable across treatments and time points (**Figure 2D**). Under 0% iron, RA23 maintained a leaf count of approximately 1.8–2 leaves, while LM displayed slightly lower values (1.4–1.9 leaves), indicating minor but visible suppression of leaf emergence in the susceptible genotype. Under 100% iron, both RA23 and LM showed almost identical leaf numbers (~2 leaves) throughout the experiment, with no significant temporal variation. These results indicate that unlike chlorophyll content and organ elongation, leaf number is less sensitive to short-term iron deficiency, though a small genotype-specific effect was observed under extreme deficiency.

##### 3.2.4.1 10th day – 0% iron

By Day 10 under iron-deficient conditions, clear morphological differences emerged between LM and RA23. LM seedlings displayed pronounced stunting, with visibly shorter shoots and poorly developed root systems consisting of thin, brittle, and sparsely branched roots. The shoots appeared pale, reflecting early chlorosis and reduced vigor (**Figure 3A**).

In contrast, RA23 maintained comparatively better growth, with longer shoots and more elongated primary roots. Although symptoms of iron deficiency were visible, RA23 roots appeared thicker and more sustained, with greater overall biomass than LM. These observations indicate that RA23 can better withstand early-stage iron deprivation.

##### 3.2.4.2 10th day – 100% iron

Under iron-sufficient conditions on Day 10, both genotypes showed improved vigor relative to their deficient counterparts. LM seedlings exhibited healthier green shoots and longer roots with more uniform growth. However, RA23 consistently outperformed LM, showing taller shoots, robust leaf emergence, and longer root systems with greater lateral extension (**Figure 3D**).

The difference in absolute size between RA23 and LM was noticeable even under sufficient iron, suggesting that RA23 has inherently stronger early growth capacity, which becomes more pronounced when iron is available.

##### 3.2.4.3 15th day – 0% iron

By Day 15, iron deficiency further accentuated the contrasting tolerance levels. LM showed severe stunting, with extremely short shoots and visibly reduced leaf expansion. Roots were highly restricted, appearing short and fragile, with limited elongation and minimal branching. Symptoms of chlorosis were visually more evident compared to Day 10 (**Figure 3B**).

RA23 again showed better resilience. While iron deficiency did slow its growth, RA23 seedlings maintained greater shoot height, wider leaf angles, and more prominent root systems. Root length remained significantly higher than in LM, with visible elongation and better structural integrity. This stage strongly highlighted RA23’s capacity to maintain root growth under nutrient stress.

##### 3.2.4.4 15th day – 100% iron

At Day 15 under sufficient iron supply, both genotypes exhibited robust growth, yet RA23 continued to display superior morphology. LM seedlings grew taller and healthier than under deficient conditions, with visibly longer roots and greener shoots. However, RA23 seedlings were consistently taller, with longer and straighter shoots and thicker, well-developed roots (**Figure 3E**).

Root architecture in RA23 showed clear advantages, including increased length and uniformity compared to LM. The improved vigor under 100% iron at this time point emphasizes the importance of adequate iron for maintaining rapid vegetative growth.

##### 3.2.4.5 20th day – 0% iron

Based on the progressive trend observed at earlier time points and quantitative measurements, LM seedlings at Day 20 under 0% iron would likely exhibit severe iron deficiency symptoms: extreme chlorosis, minimal shoot elongation, and drastically reduced root length. Biomass accumulation would be minimal, with fragile, underdeveloped roots and markedly stunted shoots (**Figure 3C**).

RA23, although affected, would still maintain comparatively taller shoots, more visible leaf expansion, and longer root systems. Root elongation would persist better than in LM, reflecting RA23’s stronger tolerance to prolonged iron deprivation.

##### 3.2.4.6 20th day – 100%

By Day 20 under iron-sufficient conditions, both genotypes showed vigorous growth, but clear differences in shoot–root allocation emerged. LM exhibited taller shoots and longer leaf blades, reflecting its identity as a cultivated variety bred for rapid shoot elongation under optimal nutrients. In contrast, RA23 seedlings were shorter yet displayed longer, thicker, and more developed root systems. This pattern aligns with typical landrace strategies, where plants invest more in below-ground growth to enhance nutrient acquisition and resilience (**Figure 3F**). Thus, although LM surpassed RA23 in shoot height under 100% iron, RA23 maintained a more robust and efficient root system, highlighting distinct adaptive growth strategies.

### 3.3 Transcriptome analysis - RNA-seq quality control and mapping

#### 3.3.1 Transcript quality control

Read quality assessed with fastp showed uniformly high sequencing performance across all 12 libraries. For every sample, 95.5–97.9% of reads passed filters, with GC content tightly clustered around 48.7–50.9% and duplication rates between 31.6% and 53.6% (**Supplementary Table 3**). Adapter trimming ranged from 25.3–50.7%, indicating efficient removal of adapter contamination. Per-base quality plots for Read1 and Read2 (**Supplementary Figure 1**) showed Phred scores remaining close to or above Q30 across the full read length, confirming that the data were of sufficient quality for downstream mapping and differential expression analysis.

#### 3.3.2 Overview of transcriptome profiling and DEG statistics

##### 3.3.2.1 Differentially expressed genes across genotypes and iron treatments

**Figure 4:**
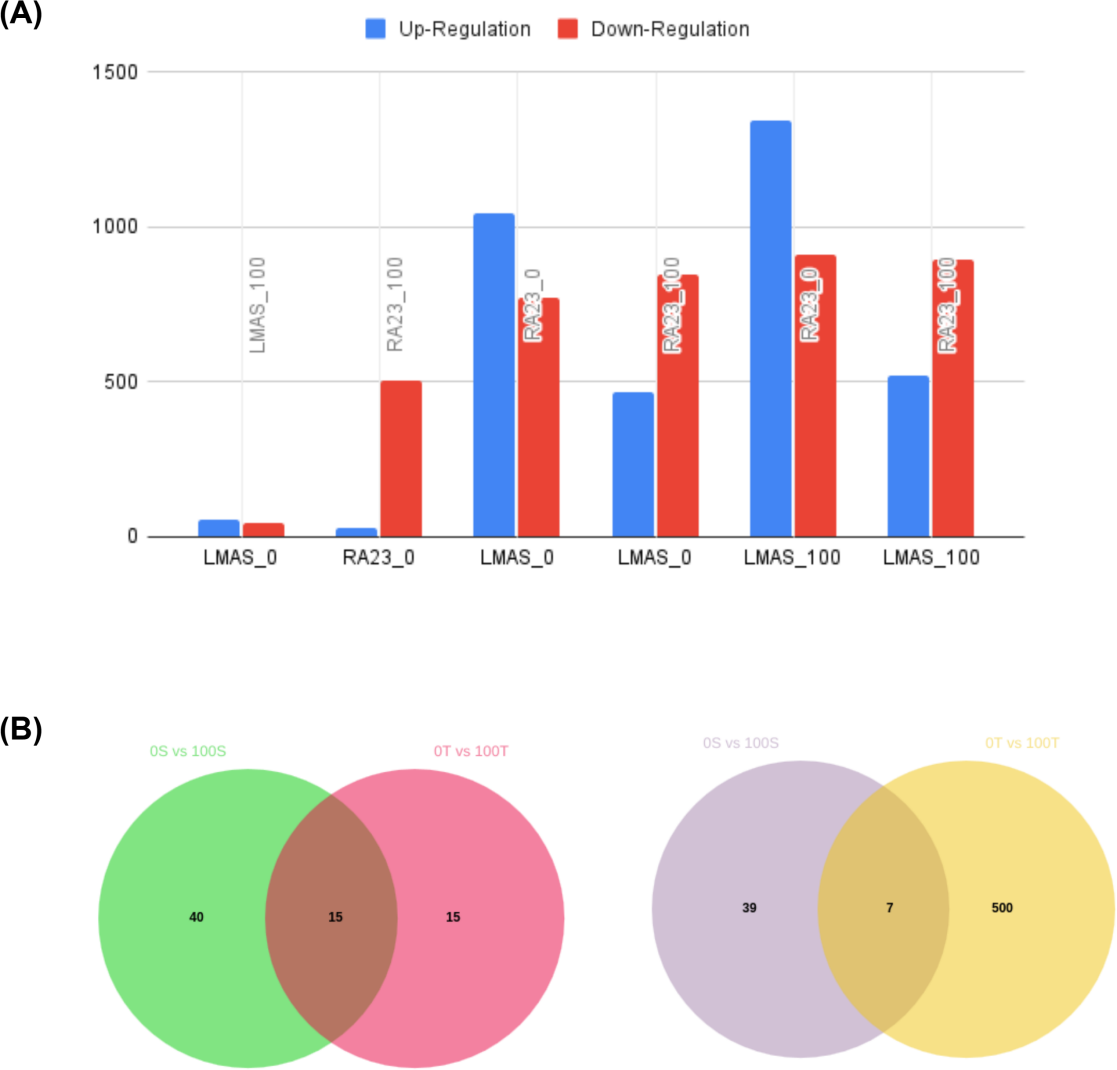
**(A)** Differentially expressed gene counts across genotype and iron-treatment comparisons. Bar plot showing the number of up- and down-regulated genes identified from pairwise contrasts between LM and RA23 under iron-deficient (0%) and iron-sufficient (100%) conditions. Intervarietal comparisons (LMAS_0 vs RA23_0; LMAS_0 vs RA23_100; LMAS_100 vs RA23_0; LMAS_100 vs RA23_100) yield substantially larger DEG sets than within-variety contrasts, highlighting strong genotype-dependent transcriptional divergence in response to iron status. **(B)** Venn diagrams showing commonly upregulated and downregulated genes under iron deficiency in LM (0S vs 100S) and RA23 (0T vs 100T); Left = Upregulated, Right = Downregulated. Forty genes were uniquely upregulated in LM, 15 in RA23, and 15 genes were commonly induced in both genotypes, representing the shared core iron-responsive module activated under 0% iron conditions. LM exhibited 39 uniquely downregulated genes, whereas RA23 showed 500 unique downregulated genes. Only seven genes were commonly repressed in both genotypes, indicating largely genotype-specific transcriptional suppression in response to 0% iron treatment.

DEG counts (**Figure 4A**; **Supplementary File 3**) reveal pronounced genotype- and treatment-dependent transcriptional differences. Within-variety contrasts were modest for LM (LMAS_0 vs LMAS_100: 55 up) but larger for RA23 (RA23_0 vs RA23_100: 30 up, 507 down). Intervarietal comparisons showed substantial divergence: LMAS_0 vs RA23_0 exhibited 1,045 up and 771 down genes, while LMAS_0 vs RA23_100 had 466 up and 847 down. Comparisons of sufficiency/baseline across genotypes were dominated by induction in LMAS_100 vs RA23_0 (1,346 up, 912 down) and LMAS_100 vs RA23_100 (520 up, 894 down). Overall, these results indicate that intervarietal contrasts account for the bulk of transcriptional variation, with the largest DEG sets arising from genotype-to-genotype comparisons rather than within-variety treatment responses.

###### 3.3.2.1.1 Comparison across varieties

The Venn diagram shows that 15 genes are commonly upregulated in both LM (0S vs 100S) and RA23 (0T vs 100T) when seedlings are exposed to iron deficiency (0%) relative to iron-sufficient controls (100%). This indicates that both varieties activate a shared core iron-responsive pathway under stress. Functional annotation of these 15 genes reveals that they include key iron-homeostasis regulators such as *OsIMA1, OsIRO2, OsIRO3, OsYSL2*, and *OsFRO2*, all of which are central components of the iron-uptake and mobilization network. Notably, although both varieties induce these canonical iron-responsive genes at 0%, LM exhibits a higher number of uniquely upregulated genes than RA23, suggesting a stronger but more reactive transcriptional response in the susceptible genotype, whereas RA23 relies more on a stable core set of iron-regulatory genes. This conserved set of 15 genes therefore represents the fundamental iron-deficiency response module shared across both genotypes (**Supplementary File 3**; **Figure 4B**). Out of these genes, 6 genes have been validated through rt-PCR.

##### 3.3.2.2 PCA reveals strong genotype-driven clustering of transcriptomes

**Figure 5:**
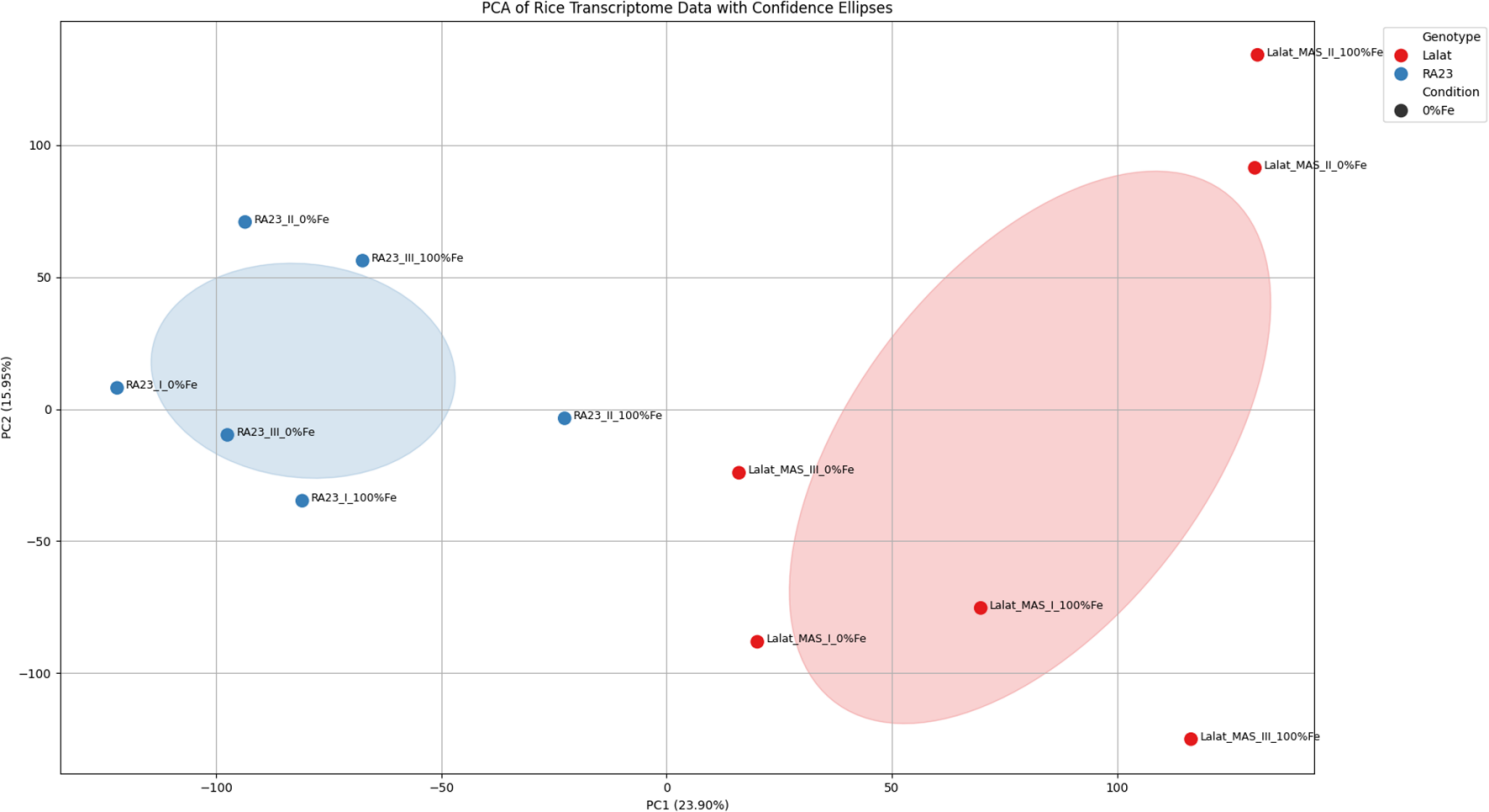
Principal component analysis (PCA) showing genotype-driven clustering of transcriptomes in RA23 and LM. PCA of global gene expression profiles reveals strong separation between RA23 (blue) and LM (red) along PC1, with RA23 forming a compact, stable cluster and LM displaying broader dispersion. Confidence ellipses highlight greater transcriptional stability in the tolerant genotype (RA23) and higher variability in the susceptible genotype (LM), indicating pronounced genotype-dependent transcriptomic divergence under iron treatments.

Principal component analysis (PCA) of the global transcriptome dataset demonstrated a clear genotype-dependent separation between LM and RA23 (**Figure 5**). PC1 (23.90%) distinctly segregated the two genotypes, with all RA23 replicates forming a compact cluster on the negative axis and all LM replicates grouping on the positive axis, independent of iron treatment. PC2 (15.35%) captured additional variation associated with iron status and biological replicates. The tight clustering of RA23 samples, reflected by a narrow confidence ellipse, indicates high transcriptional stability within the tolerant genotype, whereas LM exhibited broader dispersion, suggesting greater variability in its transcriptional response to iron availability. Together, these patterns confirm that genotype is the primary driver of global transcriptomic variation and support subsequent differential expression analyses. Overall, the PCA highlights strong genotype-driven transcriptomic divergence, validating that genotype is the dominant source of variation and supporting downstream differential expression analyses.

### 3.4 Candidate gene prioritization and novel candidate identification

#### 3.4.1 Candidate gene prioritization

Candidate genes were prioritized using an integrated workflow combining differential expression, annotation, and structural inference. Significant DEGs from volcano plots were retrieved from RAP-DB and assessed in STRINGdb to identify interaction partners and links to iron-related pathways. For genes lacking STRINGdb information, sequence- and structure-based analyses (PSI-BLAST, HHPred, Foldseek, AlphaFold) were used to detect remote homology.

##### 3.4.1.1 Volcano plots show the outliers

###### 3.4.1.1.1 LM vs RA23 (0S vs 0T)

**Figure 6:**
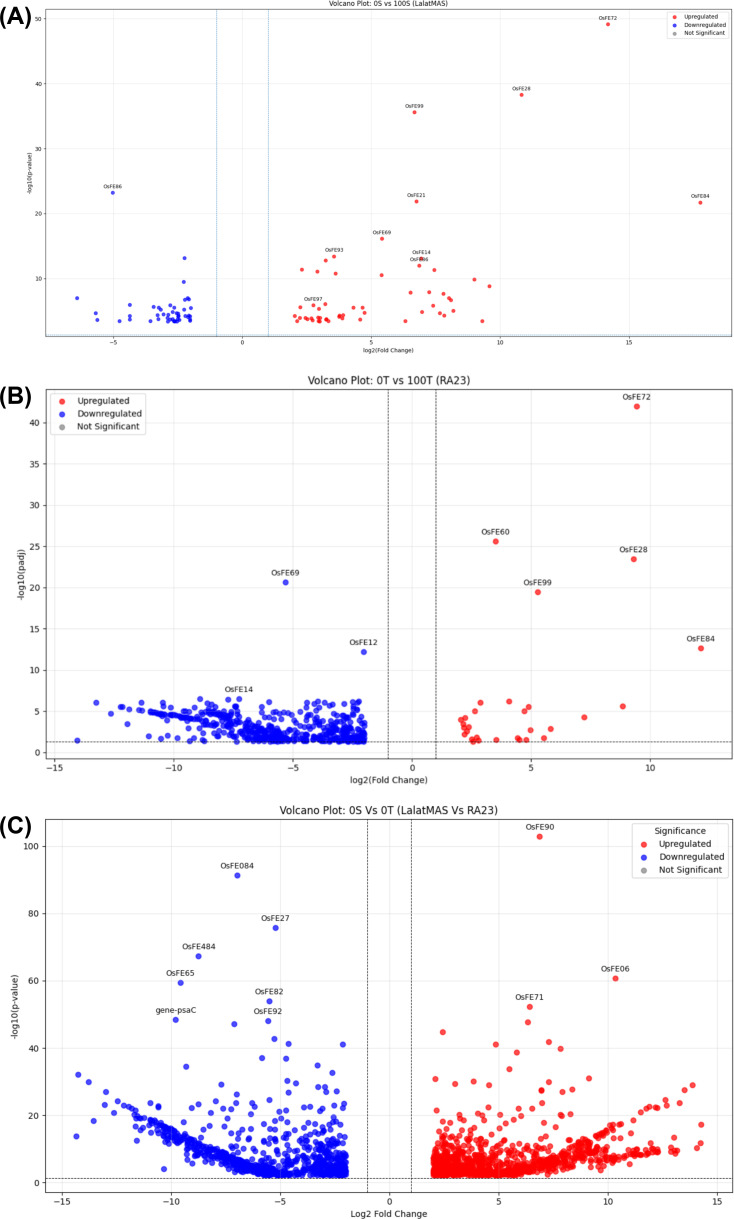
**(A)** Volcano plot showing differentially expressed genes between LM (0S) and RA23 (0T) under iron-deficient (0% iron) conditions. Strongly upregulated (red) and downregulated (blue) genes highlight pronounced genotype-specific transcriptional responses, with LM displaying sharper induction and repression relative to the more stable expression profile of RA23. **(B)** Volcano plot showing differentially expressed genes in LM under iron-deficient conditions (0S vs 100S). A strong induction of canonical iron-responsive genes—including O*sIMA1, OsIRO2, OsYSL2, OsNRAMP1*, and *OsFRO2*—is observed on the upregulated side, reflecting activation of iron uptake and mobilization pathways. A smaller cluster of strongly downregulated genes, such as OsFE69, indicates transcripts suppressed during iron deficiency. **(C)** Volcano plot showing differentially expressed genes in RA23 under iron deficiency (0T vs 100T). Core iron-homeostasis genes (OsIMA1, OsIRO2, OsYSL2, OsNRAMP1, OsFRO2) are prominently upregulated, similar to LM. RA23 exhibits a broader and denser distribution of downregulated genes, indicating stronger transcriptional repression of non-essential pathways as part of its tolerance strategy.

The volcano plot illustrates the transcriptomic differences between LM (0S) and RA23 (0T) under identical iron-deficient conditions (0% iron) (**Figure 6A**). A substantial number of DEGs were detected, reflecting distinct stress-response strategies in the two genotypes. Several genes showed extreme fold changes, indicating strong genotype-specific regulation. A large set of downregulated genes (blue) in LM relative to RA23 includes highly suppressed transcripts such as OsFE84, OsFE27, OsFE82, OsFE92, and the photosystem gene psaC, suggesting reduced photosynthetic activity and metabolic downscaling in the susceptible genotype. Conversely, the upregulated genes (red) in LM relative to RA23 include loci such as OsFE50, OsFE71, and OsFE06, representing strongly induced stress-related or compensatory pathways. Overall, the volcano plot indicates that LM exhibits a more reactive and sharply polarized expression pattern, with both strong repression and strong induction of genes, whereas RA23 maintains a more stable transcriptomic profile under iron deficiency. This pattern reinforces RA23’s tolerant phenotype and LM’s heightened sensitivity to iron deficiency. The outliers were further picked for annotation.

###### 3.4.1.1.2 LM (0S vs 100S)

The volcano plot for LM shows a strong iron-deficiency–induced transcriptional activation, with a large set of significantly upregulated genes (right side, red) and fewer downregulated genes (left side, blue) (**Figure 6B**). Several well-established iron-responsive regulators, including *OsIMA1* (OsFE72), *OsIRO2* (OsFE28), *OsYSL2* (OsFE99), *OsNRAMP1* (OsFE84), and *OsFRO2* (OsFE86) appear as prominent upregulated DEGs, indicating robust activation of iron uptake, transport, and signaling pathways in the susceptible genotype during iron deficiency. A smaller set of strongly downregulated genes such as OsFE69 was also observed, representing transcripts repressed under 0% iron.

###### 3.4.1.1.3 RA23 (0T vs 100T)

In RA23, a similar set of canonical iron-responsive genes showed significant upregulation under 0% iron when compared with 100% iron (**Figure 6C**). The same core iron-regulated genes—*OsIMA1, OsIRO2, OsYSL2, OsNRAMP1, OsFRO2*—were also strongly induced in the tolerant genotype. However, RA23 displayed a larger and more pronounced cluster of downregulated genes, suggesting that the tolerant genotype may suppress a broader set of non-essential processes to conserve resources under iron deprivation. The downregulated region was more densely populated than in LM, consistent with stronger transcriptional repression in RA23.

##### 3.4.1.2 Common signature between LM and RA23

A notable observation is that the major iron homeostasis regulators are consistently upregulated in both genotypes, highlighting a shared conserved iron-deficiency response module. Genes such as: *OsIMA1* (Fe signaling), *OsIRO2* (master bHLH transcription factor), *OsYSL2* (nicotianamine-mediated Fe transporter), *OsNRAMP1* (Fe/Mn transporter), *OsFRO*2 (Fe(III) reductase) all appear prominently in both volcano plots, confirming their central role in iron acquisition and mobilization under iron deficiency. Despite this shared core response, LM showed stronger upregulation, while RA23 displayed broader and deeper downregulation, reflecting susceptibility- vs. tolerance-linked strategies, respectively.

#### 3.4.2 Novel candidate identification

Outlier genes identified from volcano plots were first annotated using RAP-DB (**Supplementary File 3**), which enabled separation of known iron-related genes from poorly annotated or hypothetical loci. From this refined set, several transcripts lacking clear functional information emerged as strong novel candidates. While some of these genes showed interaction links in STRINGdb, few lacked any PPI evidence, prompting deeper investigation. These candidates were subsequently analyzed using PSI-BLAST, HHPred, Foldseek, AlphaFold, and TMHMM (**Supplementary File 4**), which collectively revealed biologically meaningful sequence homology, conserved structural motifs, and transmembrane helix predictions, together suggesting that these proteins may belong to metal-associated or membrane-linked functional classes. Notably, this paper presents detailed results of PPI analysis, PSI-BLAST homology search, and HHPred-based structural predictions for a single representative candidate gene (which has also been validated further as an iron responsive gene in qRT-PCR).

##### 3.4.2.1 Protein–protein interaction network analysis

**Figure 7:**
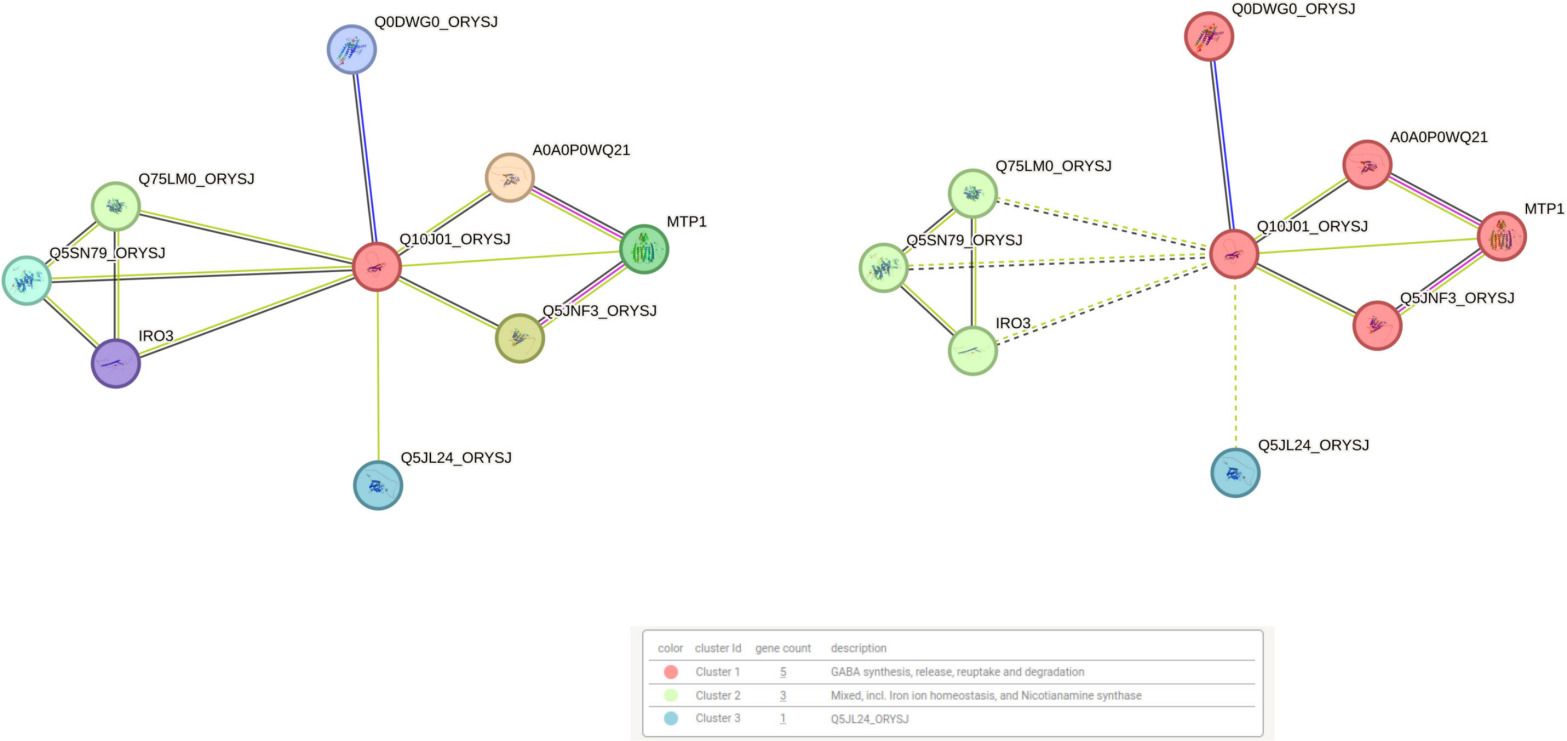
Protein–protein interaction (PPI) network and functional annotation of the hypothetical gene OsFE97 (DUF1230 family). STRING analysis shows its associations with proteins involved in GABA metabolism, iron homeostasis, and nicotianamine synthesis. Interaction with MTP1, a metal tolerance protein, suggests a potential functional role for OsFE97 in metal ion regulation and stress adaptation.

To investigate the possible function of the hypothetical gene OsFE97 (DUF1230 family), RAP-DB annotation and STRING-based protein interaction analyses were performed (**Figure 7**). InterPro domain search identified a CGLD27-like (IPR009631) domain, previously associated with metal ion homeostasis in plants. The STRING PPI network revealed OsFE97 interaction clusters with proteins enriched in GABA metabolism, iron homeostasis, and nicotianamine synthesis clusters. Notably, the network contained MtP1, a metal tolerance protein involved in zinc sequestration and cellular metal homeostasis, suggesting potential functional linkage of OsFE7 with metal transport or stress-response pathways. Although OsFE97 remains functionally uncharacterized, its PPI interactions, CGLD27-like domain architecture, and qRT-PCR validation all support a potential role in metal-associated processes, particularly iron homeostasis under stress (0S vs 100S). Furthermore, literature evidence shows that CGLD27 orthologs—including the rice orthologue OsFE97—are conserved across plastid-containing organisms and share homology with the algal plastid open reading frame ycf36 (Douglas and Penny, 1999), suggesting chloroplast targeting and possible involvement in chloroplast-associated metal regulation.

##### 3.4.2.2 OsFE97 emerges as a novel candidate gene in 0s vs 100s: sequence, structure, and homology characterization

###### 3.4.2.2.1 Evolutionary conservation and functional clues from PSI-BLAST

PSI-BLAST analysis revealed that OsFE97, although annotated as a DUF1230 family protein, is conserved across diverse plant and algal species, several of which exhibit iron-responsive behavior—including orthologs in *Arabidopsis thaliana* and *Chlamydomonas reinhardtii*. Structural and domain analysis further indicated that OsFE97 belongs to the CGLD27-like family, a highly conserved plastid-associated protein group predicted to contain three transmembrane helices and commonly targeted to the chloroplast. Literature reports identify its orthologs (e.g., *A. thaliana* At5g67370) as plastid proteins related to the algal open reading frame ycf36, which is implicated in stress-associated plastid functions.

Comparative expression data from *Arabidopsis* show that CGLD27 exhibits a spatial expression pattern resembling FRO3, a ferric-reductase involved in iron homeostasis, further supporting its potential association with metal-related processes (Eugenio et al., 2012). Consistent with this, OsFE97 in our study was strongly upregulated under iron deficiency and validated by qRT-PCR, reinforcing its status as a previously uncharacterized iron-responsive gene with a likely role in iron–zinc homeostasis or plastidial metal-associated stress signaling.

###### 3.4.2.2.2 High-confidence homology support from PSI-BLAST iterative searches

The second-iteration PSI-BLAST search for OsFE97 retrieved an extensive set of highly conserved homologous sequences predominantly annotated as “proteins conserved in the green lineage and diatoms,” in addition to several hypothetical or uncharacterized proteins from diverse plant species. These hits showed exceptionally strong statistical support, with E-values ranging from 1e-153 to 1e-146, high query coverage (>80%), and 100% positive selection across all 500 retrieved sequences. The recurrence of chloroplast-associated and green-lineage-specific proteins across taxa indicates that OsFE97 belongs to a deeply conserved plastid-linked protein family, despite the absence of detailed functional annotation in rice.

###### 3.4.2.2.3 Conserved transmembrane topology revealed by TMHMM

**Figure 8:**
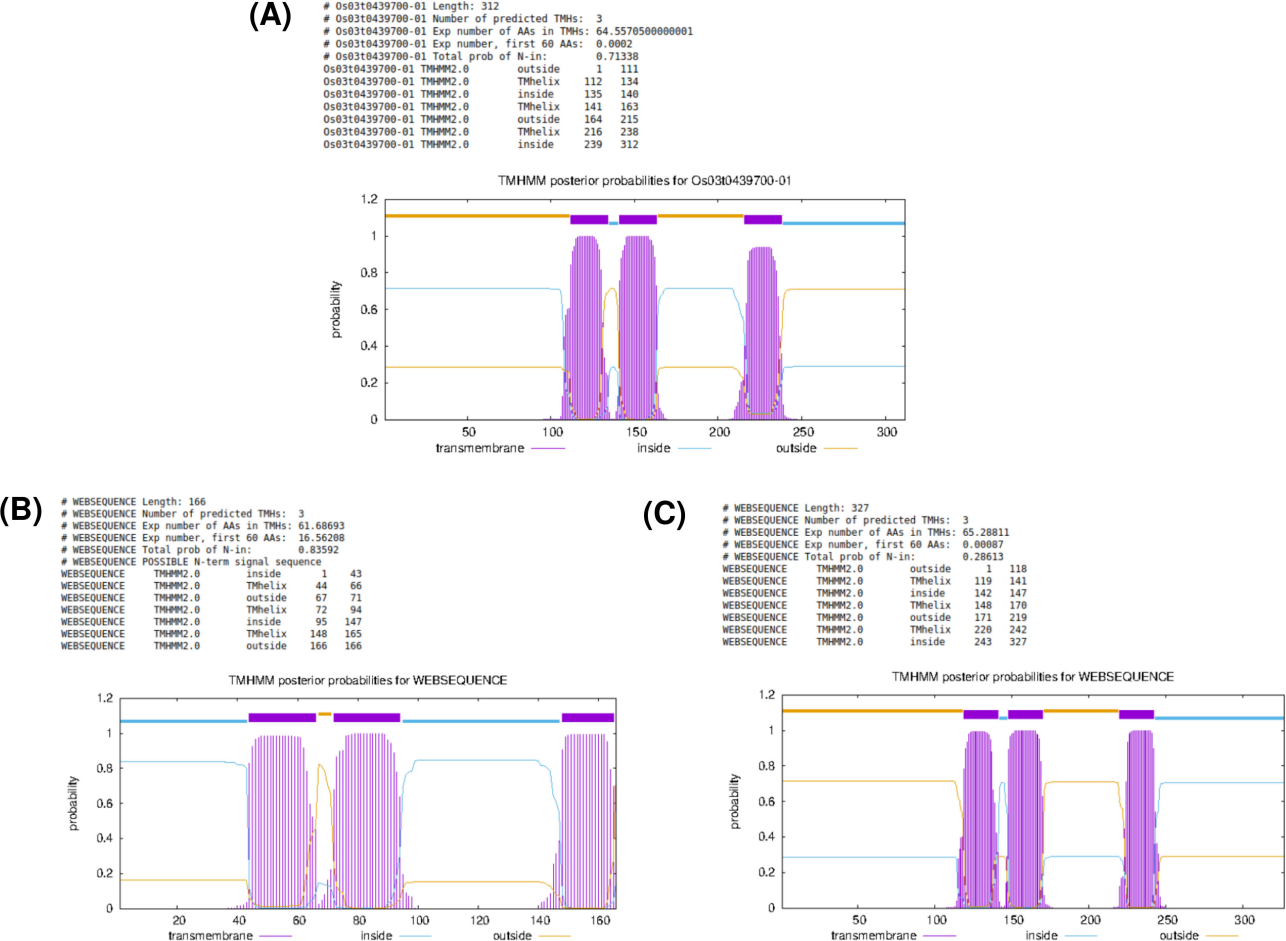
TMHMM transmembrane topology predictions for **(A)** the rice CGLD27 family protein (OsFE97), **(B)** a homolog from *Trichocoleus* sp. FACHB-69, and **(C)** the *Arabidopsis thaliana* protein At5g67370. All three sequences display a conserved pattern of three transmembrane helices with similar orientation and spacing, highlighting a shared membrane-associated architecture across monocot, dicot, and cyanobacterial lineages.

TMHMM-based transmembrane topology predictions were generated for the rice CGLD27 protein (OsFE97), its homolog from *Trichocoleus* sp. FACHB-69, and the *A. thaliana* ortholog At5g67370 (**Figures 8A–C**) respectively). All three proteins consistently exhibited three well-defined transmembrane helices with comparable positions and orientation. This conserved architecture across monocot, dicot, and cyanobacterial homologs strongly supports the evolutionary conservation of the CGLD27-like protein family and reinforces the likelihood that OsFE97 performs a membrane-associated functional role, potentially linked to plastidial metal transport or stress signaling.

###### 3.4.2.2.4 Network-level evidence supports a role for OsFE97 in iron deficiency responses

**Figure 9:**
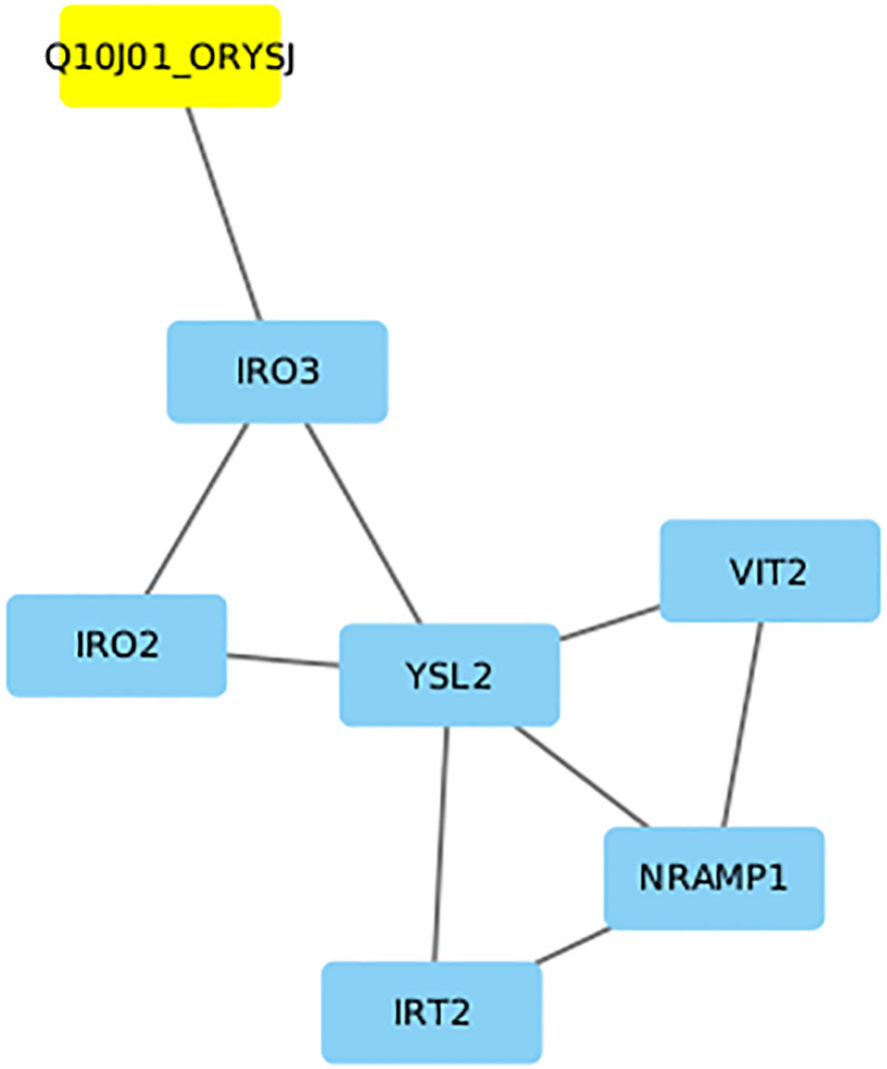
Predicted interaction network positioning OsFE97 (Q10J01_ORYSJ; highlighted in yellow) in proximity to key iron homeostasis regulators in rice. The candidate shows a direct association with IRO3, with indirect connections to IRO2, YSL2, NRAMP1, VIT2, and IRT2, forming a functional module comprising major transcriptional and transporter components involved in iron uptake, translocation, and storage.

Cytoscape-based interaction analysis positioned OsFE97 adjacent to a central regulatory module governing iron homeostasis in rice (**Figure 9**). The candidate gene showed a direct predicted interaction with IRO3, a key bHLH transcriptional repressor that modulates iron-deficiency signaling. Through IRO3, OsFE97 was indirectly connected to IRO2, a major transcriptional activator of iron uptake, and to YSL2, a metal–nicotianamine transporter facilitating long-distance iron translocation. NRAMP1, another downstream node in the cluster, mediates Fe^2+^ and Mn^2+^ influx across membranes. VIT2 (OsFE69), a vacuolar iron transporter responsible for sequestering excess iron, also appeared in the extended subnetwork but did not directly interact with OsFE97; importantly, VIT2 was downregulated in our dataset, consistent with reduced vacuolar iron storage during deficiency. IRT2 was similarly part of the peripheral transport cluster but lacked a direct edge to OsFE97. The position of OsFE97 within this transcription–transport interaction module suggests that this previously uncharacterized gene may participate in membrane-associated aspects of iron sensing, allocation, or signaling.

### 3.5 GO enrichment and pathway analysis reveal genotype-specific iron responses

#### 3.5.1 0s vs 100s — GO enrichment for combined up- and down-regulated genes for susceptible variety LM (S)

**Figure 10:**
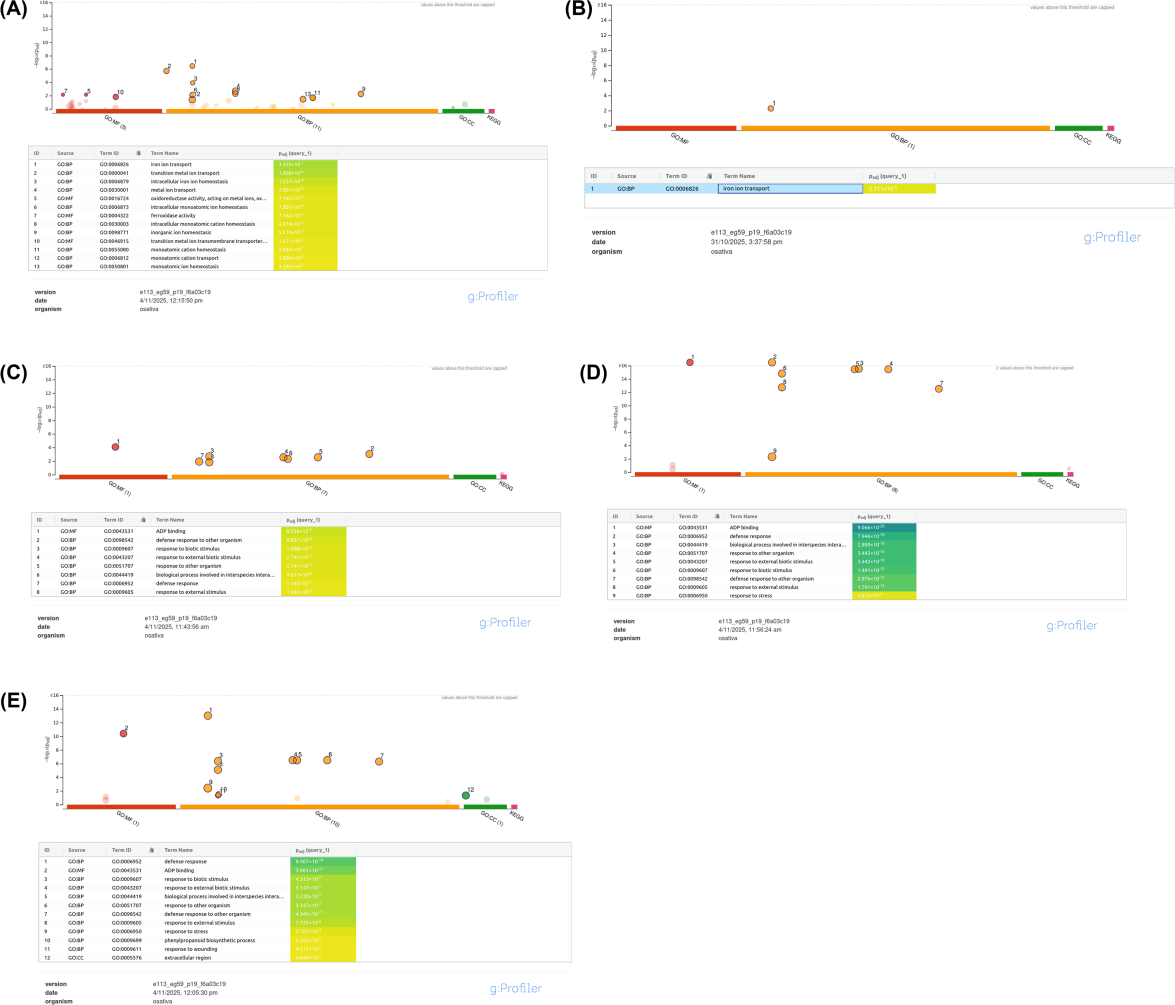
**(A)** GO enrichment dot plot analysis (g:Profiler) of the combined up-regulated and down-regulated genes from the 0s_100s comparison. Significantly enriched GO terms were predominantly associated with iron and transition-metal transport, intracellular metal homeostasis, and oxidoreductase activity. The top enriched category was *iron ion transport* (GO:0006826), followed by additional metal-handling and monoatomic cation homeostasis processes, indicating a strong metal-responsive signature in the DEG set. **(B)** GO enrichment dot plot analysis of up-regulated genes from the 0s_100s comparison. Only a single term, *iron ion transport* (GO:0006826), reached statistical significance, reflecting the limited size of the UR gene set. This enrichment nevertheless highlights that iron-transport–related processes are specifically induced in the up-regulated subset. **(C)** GO enrichment dot plot analysis (g:Profiler) of the combined up- and down-regulated genes from the 0T vs 100T comparison. Key enriched categories include response to biotic stimulus, external biotic stimulus, defense response, and stress-related processes. **(D)** GO enrichment dot plot for the combined UR+DR gene set from the 0S_vs_0T comparison, highlighting significant enrichment of defense-, biotic stimulus-, and general stress-response processes. **(E)** GO enrichment dot plot for the up-regulated gene set from the 0S_vs_0T comparison, showing significant induction of defense, external stimulus, and wounding-related biological processes.

GO over-representation analysis (g:Profiler; expressed gene universe; FDR correction) of the combined set of up- and down-regulated genes from the 0s_100s comparison returned a coherent metal-related signature (**Figure 10A**). Thirteen GO terms reached significance, dominated by ion transport and metal homeostasis categories: the top term was iron ion transport (GO:0006826; padj = 3.43×10^-7^), followed by transition metal ion transport, intracellular iron ion homeostasis, and broader metal ion transport and monoatomic cation transport/homeostasis terms. Molecular-function terms related to oxidoreductase/ferroxidase activities were also enriched, consistent with iron redox cycling. Collectively, these results indicate that the differentially expressed genes (UR + DR) in 0s_100s are strongly biased toward iron/metal transport and redox processes, supporting a coordinated metal-homeostasis response under the experimental condition.

#### 3.5.2 0s_100s — GO enrichment for up-regulated genes only for susceptible variety LM (S)

When enrichment was restricted to the up-regulated gene list alone, the analysis returned a single significant term: iron ion transport (GO:0006826; padj = 5.11×10^-3^) (**Figure 10B**). No additional transport or redox categories passed FDR for the UR subset. This more limited result likely reflects reduced statistical power attributable to the small UR gene list in the 0s_100s dataset: while the iron-transport signal is still detectable among induced genes, several related processes (e.g., transition metal transport, oxidoreductase activity) only reached significance when UR and DR genes were considered together.

#### 3.5.3 0T_vs_100T – GO enrichment results for combined up- and down-regulated genes for tolerant variety RA23 (S)

Because the 0T_vs_100T comparison yielded only 30 up-regulated genes, the UR set alone did not produce any significant enrichment in g:Profiler. To obtain statistically meaningful results, the up- and down-regulated genes were combined and analyzed together using the expressed-gene background. This combined analysis revealed nine significantly enriched GO terms, predominantly associated with defense- and stress-related pathways. The top terms included response to biotic stimulus (GO:0009607; padj = 3.58×10^-5^) (**Figure 10C**), response to external biotic stimulus, defense response to other organisms, and response to stress, indicating activation of generalized stress-response mechanisms under the 100T condition. A single molecular function category, ATP binding (GO:0043531), also appeared among the significant terms. These results suggest that, unlike the iron-transport enrichment observed in other comparisons, the 0T vs 100T contrast is characterized primarily by broad biotic and external-stimulus response pathways, reflecting a distinct stress-associated transcriptional signature.

#### 3.5.4 0S vs 0T – GO enrichment results for combined up- and down-regulated genes for both S and T

GO enrichment of the combined up- and down-regulated genes from the 0S vs 0T comparison revealed a strong activation of defense- and stimulus-related pathways. Nine GO terms passed the significance threshold, dominated by defense response (GO:0006952; padj = 7.95×10^-19^) (**Figure 10D**), biological process involved in interspecies interaction, response to biotic stimulus, and response to external biotic stimulus. Additional enriched categories included response to other organisms, response to external stimulus, and response to stress, collectively indicating broad activation of biotic-interaction and stress-response(both abiotic and biotic) programs at the 0T stage. One molecular function term, ADP binding, was also significantly enriched. This enrichment profile suggests that early-stage transcriptomic differences between seedlings at 0S and 0T involve generalized biotic and environmental stimulus responses.

#### 3.5.5 0S vs 0T – GO enrichment results for only up-regulated genes for both S and T

GO enrichment of the up-regulated genes from the 0S_vs_0T comparison revealed ten significant terms, primarily associated with defense and biotic stress responses. The strongest category was defense response (GO:0006952; padj = 9.40×10^-14^) (**Figure 10E**), followed by response to biotic stimulus, response to external biotic stimulus, and biological processes involved in interspecies interactions. Additional enriched terms included response to other organisms, response to external stimulus, response to stress, and response to wounding, indicating that the transcriptional induction at 0T is dominated by pathways associated with early defense and external-stimulus perception. A single MF term (ADP binding) and one CC category (extracellular region) were also significantly enriched.

### 3.6 Quantitative real-time PCR validation

#### 3.6.1 Candidate gene discovery and classification across datasets

To prioritize genes likely contributing to iron deficiency responses, differentially expressed genes (DEGs) were classified based on their expression patterns, annotation, predicted structure, and interaction with iron-related pathways. This classification was done for two major comparisons:

- 0S vs 0T: LM vs RA23 under 0% iron (genotype-specific iron response)
- 0S vs 100S: Fe-deficient vs Fe-sufficient in LM (treatment-specific iron response)
- 0T vs 100T: Fe-deficient vs Fe-sufficient in RA23 (treatment-specific iron response)

#### 3.6.2 qRT-PCR validation of iron-responsive candidate genes

##### 3.6.2.1 Iron-induced intravarietal expression dynamics in LM (0S vs 100S)

**Figure 11:**
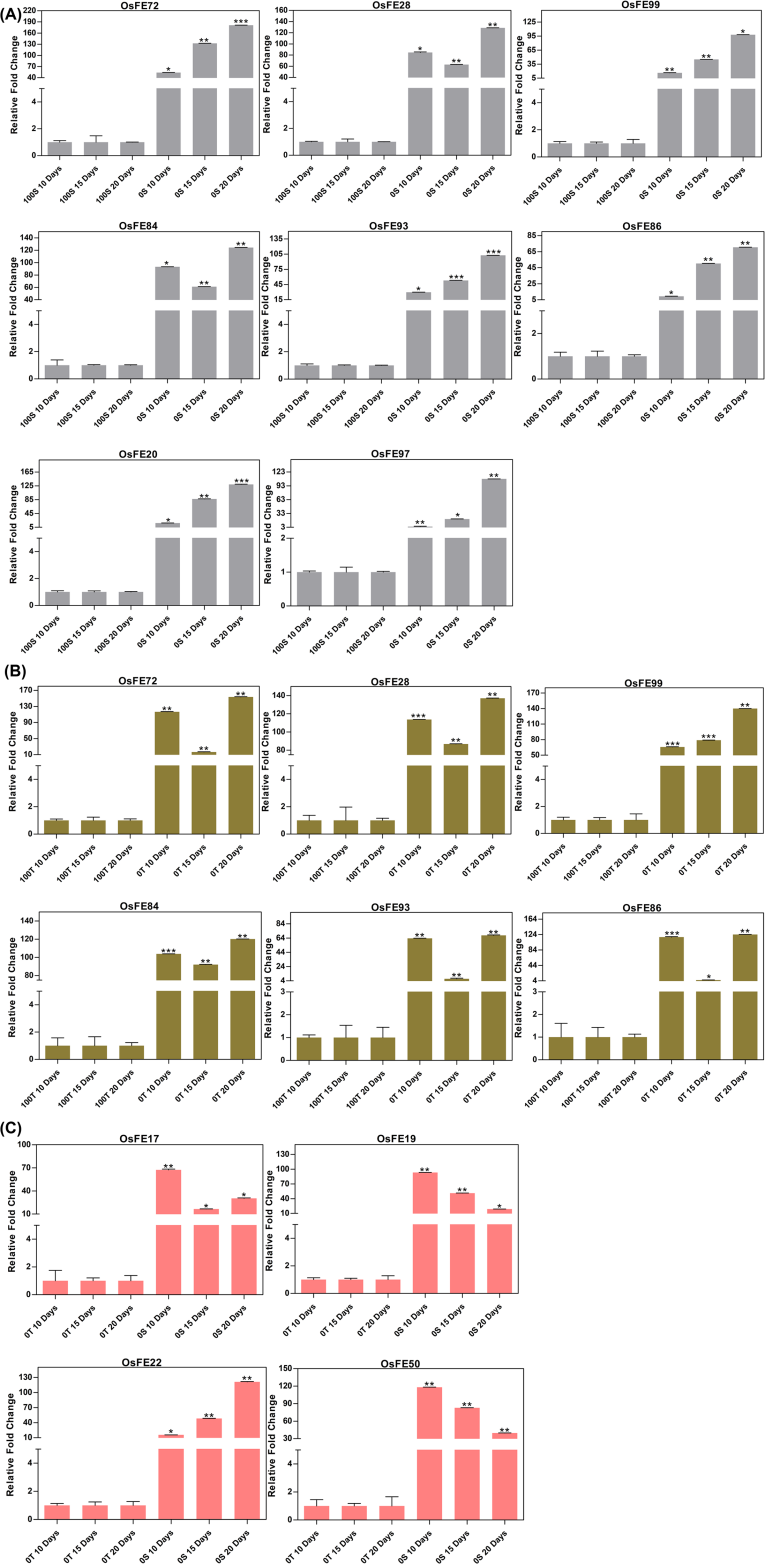
**(A)** Iron-induced intravarietal expression dynamics in LM (0S vs 100S) - qRT-PCR analysis showing expression profiles of eight iron-responsive genes in LM under control (0S) and iron-treated (100S) conditions across 10, 15, and 20 days. Control samples exhibited consistently low transcript levels, while treated seedlings showed progressive, mostly monotonic upregulation, with several genes peaking at Day 20. The hypothetical gene OsFE97 displayed a strong time-dependent induction, identifying it as a key novel iron-responsive candidate in the susceptible genotype. **(B)** Iron-induced intravarietal expression dynamics in RA23 (0T vs 100T) - Expression patterns of candidate genes in the tolerant genotype RA23 across control (0T) and iron-treated (100T) seedlings over 10, 15, and 20 days. Control samples remained uniformly low, whereas treated samples exhibited robust and sustained induction, with the highest expression often observed at Day 20. The strong transcriptional activation under iron sufficiency reflects RA23’s enhanced iron-use efficiency and tolerant phenotype. **(C)** Intervarietal expression patterns under iron-deficient conditions (0S vs 0T) - Comparative qRT-PCR profiles of four candidate genes in LM (0S) and RA23 (0T) grown under iron-deficient conditions. Control samples showed low and stable expression across all time points, while treated samples demonstrated clear time-dependent increases, with stronger induction at later stages. The hypothetical Kelch-domain gene OsFE22 followed this characteristic trend, supporting its identification as a novel iron-responsive candidate in the intervarietal comparison. The asterisks ***, **, and * denote statistical significance at p-values of <0.0001, <0.001, and <0.05, respectively.

In the susceptible genotype, the first three (control) bars remained low and largely unchanged over time, whereas the treated samples showed marked, mostly monotonic increases from Day 10 to Day 20 for nearly all eight genes. Several genes reached their highest expression at Day 20, indicating sustained activation during prolonged treatment, with only minor deviations from this general increasing trend. Among them, OsFE97, a hypothetical, globular protein mirrored this pattern of minimal control expression and strong, time-dependent upregulation, highlighting it as a key novel candidate from the 0S_100S dataset (**Figure 11A**).

##### 3.6.2.2 Iron-induced intravarietal expression dynamics in RA23 (0T vs 100T)

A similar control–treatment contrast was observed in the tolerant genotype, but with generally higher fold changes in the treated samples. Control bars remained low across all time points, whereas treated seedlings showed robust increases in expression, often with the largest induction at Day 20. This indicates a strong and sustained transcriptional response in the tolerant background, consistent with its enhanced iron-use efficiency (**Figure 11B**).

##### 3.6.2.3 Intervarietal expression patterns under iron-deficient conditions (0S vs 0T)

For all four gene transcripts (OsFE17, OsFE19, OsFE22 and OsFE50), control samples showed low and relatively stable expression across 10, 15 and 20 days. However, the treated seedlings displayed a clear time-dependent variability in transcript abundance, with higher fold changes at Day 10 compared to Day 15 for most genes. This pattern indicates progressive activation of these loci under the 0S_0T condition. Notably, OsFE22, annotated as a hypothetical Kelch-domain protein, followed the same trend of low control expression and strong induction over time, supporting its designation as a novel iron-responsive candidate in this comparison (**Figure 11C**).

#### 3.6.3 qRT-PCR validation confirms RNA-seq expression trends

**Figure 12:**
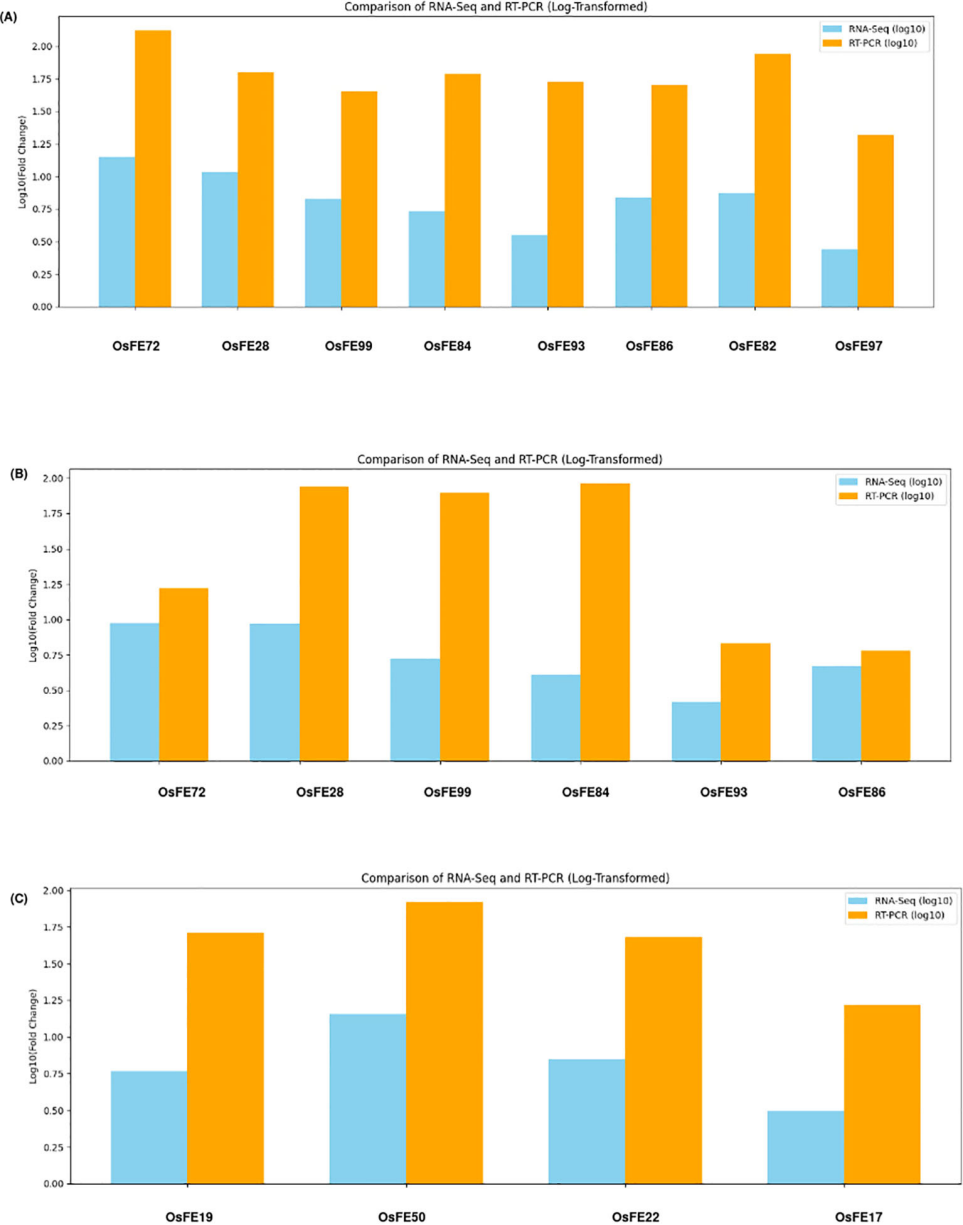
**(A)** qRT-PCR validation of 8 selected DEGs showing correlation with RNA-seq expression patterns (0S Vs 100S). **(B)** qRT-PCR validation of 6 selected DEGs showing correlation with RNA-seq expression patterns (0T VS 100T). **(C)** qRT-PCR validation of 4 selected DEGs showing correlation with RNA-seq expression patterns (0S Vs 0T).

For 0S vs 100S, key iron-responsive genes—*OsIRO2* (OsFE93), *OsYSL2* (OsFE99) and *OsFRO2* (OsFE86)—showed closely matching profiles between RNA-seq and qRT-PCR, reflecting their roles in iron uptake, transport, and redox regulation. Similar agreement was observed for additional regulatory and transporter genes including *OsIMA1* (OsFE72), *OsNRAMP1* (OsFE84), *OsIRO3/BHLH63* (OsFE93), *OsMIR* (OsFE20) (**Figure 12A**). Similarly, the hypothetical DUF1230-domain DEG OsFE97 showed highly consistent expression patterns across both platforms, reinforcing its designation as a previously uncharacterized but responsive component of the iron-deficiency network, and emerges as a novel candidate. For 0T vs 100T, core iron-related genes such as *OsIMA1, OsIRO2, OsYSL2, OsNRAMP1, OsIRO3*, and *OsFRO2* again showed highly consistent induction trends across both platforms, validating their coordinated activation in the tolerant genotype and reinforcing their conserved roles in systemic iron regulation (**Figure 12B**). For 0S vs 0T, transport-associated genes *OsMTP11.1* (OsFE19) and *OsHKT1* (OsFE17) exhibited comparable upregulation in qRT-PCR and RNA-seq, reflecting their contribution to metal transport and ionic balance under iron stress (**Figure 12C**). Notably, the uncharacterized DUF-containing genes OsFE22 (DUF1677) and OsFE50 (DUF3778) also showed strong cross-platform agreement, supporting their identification as novel iron-responsive candidates.

Collectively, this cross-validation confirms the robustness of the RNA-seq dataset and underscores the biological relevance of both known and newly identified genes in shaping the iron-deficiency response in rice.

#### 3.6.4 Varietal dissimilarity - tolerance of RA23 against iron deficiency over LM

##### 3.6.4.1 Comparative expression profiling of commonly upregulated (fighter) genes

**Figure 13:**
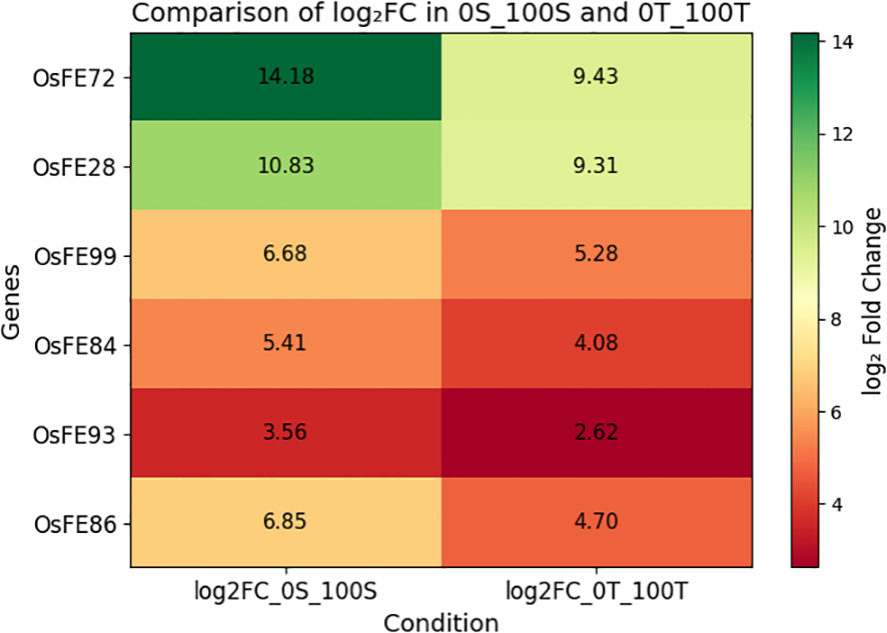
Common UR genes (significant) between 0S_100S and 0T_100T.

A subset of genes that were significantly upregulated under iron stress in both susceptible (LM; 0S vs 100S) and tolerant (RA23; 0T vs 100T) rice varieties was identified as “fighter genes.” A comparative heatmap of log_2_ fold change values demonstrated that these genes were consistently induced under iron-deficient conditions across both genotypes, indicating their central role in stress adaptation. Although all selected candidates exhibited strong upregulation (log_2_FC > 2) in both varieties, the magnitude of induction was generally higher in the susceptible line (0S_100S), suggesting a hyperactivation of defense-related transcription under severe stress (**Supplementary Table 4**, **Figure 13**). In contrast, the tolerant variety (0T vs 100T) displayed moderate yet significant induction, reflecting a more regulated and efficient stress management strategy. These consistently upregulated genes likely represent core components of the iron-deficiency response network and may contribute to basal tolerance mechanisms conserved across genetic backgrounds.

##### 3.6.4.2 Intervarietal validation of RNA-seq and RT-qPCR expression patterns

**Figure 14:**
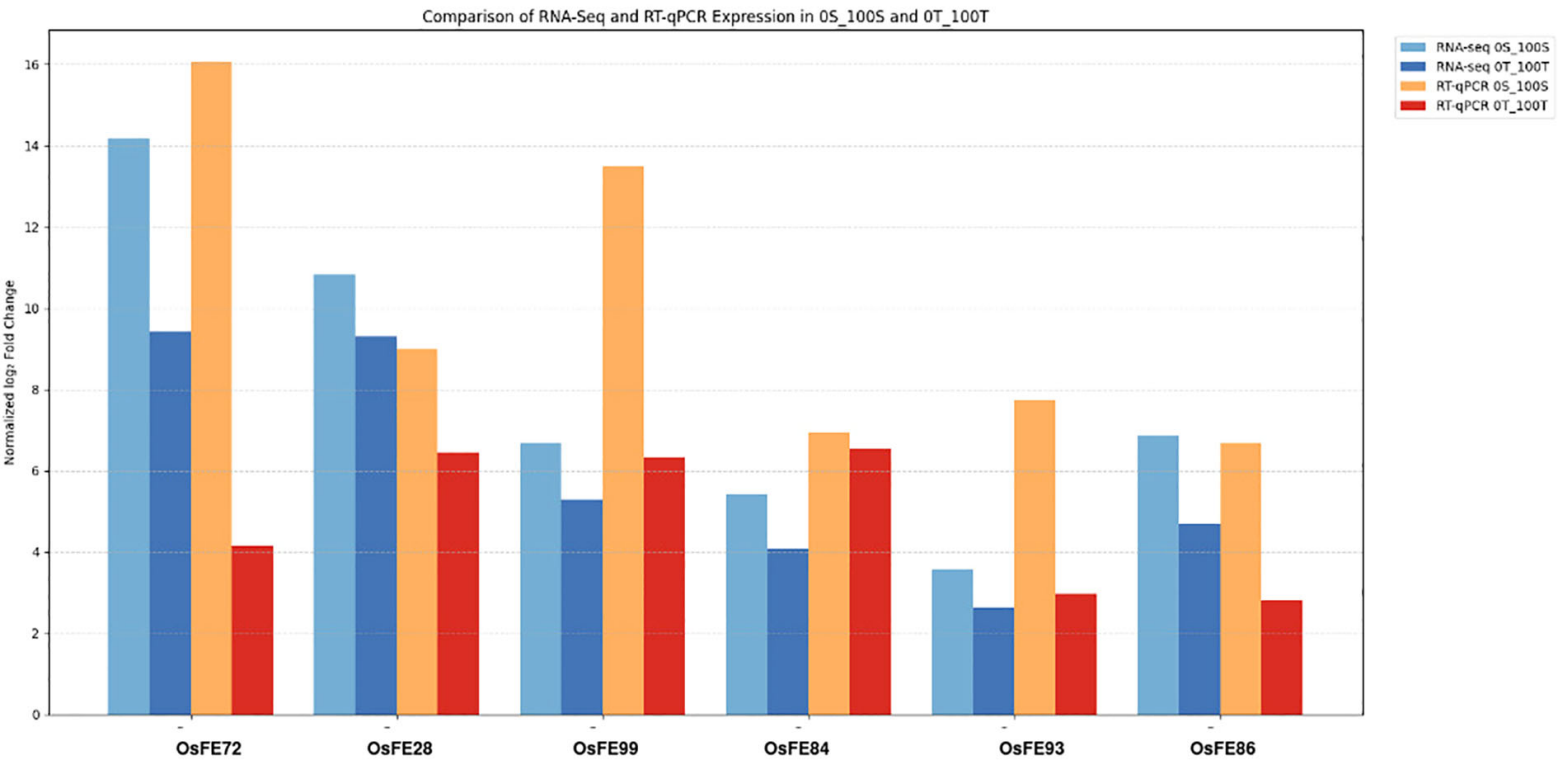
Comparison of RT-PCR and Dseq2 results amongst susceptible and tolerant variety at Iron deficient (0%) and control conditions (100%).

RNA-seq and RT-qPCR data for six iron-responsive genes (*OsIMA1, OsIRO2, OsYSL2, OsNRAMP1, OsIRO3, OsFRO2*) showed a consistent overall trend between the two platforms, namely - in LM (0S) and RA23 (0T) under 0% and 100% iron conditions. After log_2_ normalization of RT-qPCR fold-change values, expression trends closely matched RNA-Seq profiles in both varieties. Across most genes (*OsIMA1, OsIRO2, OsYSL2, OsIRO3, OsFRO2*), expression levels were higher in 0S vs 100S than 0T vs 100T, consistent with the expected “fighter gene” pattern where susceptible genotypes show stronger induction under stress or recovery conditions (**Figure 14**). *OsNRAMP1* showed similar expressions between varieties. Overall, the agreement between platforms confirms the reliability of the RNA-Seq dataset and highlights clear intervarietal differences in transcriptional responses to iron availability.

## 4 Discussion

### 4.1 Phenotypic divergence reflects inherent tolerance differences

The contrasting phenotypic responses of RA23 and LM to iron deficiency clearly demonstrate their differential adaptive capacities. RA23 consistently maintained higher SPAD values, greater biomass accumulation, and a more vigorous root system compared with LM. These traits align well with documented characteristics of iron-efficient rice genotypes, which sustain chlorophyll content, root elongation, and shoot vigor under iron-limited conditions through enhanced acquisition and optimized internal translocation (Kobayashi and Nishizawa, 2012; Bashir et al., 2013). In contrast, the rapid chlorosis, reduced growth, and compromised root development observed in LM are typical of iron-inefficient cultivars, where low Fe uptake capacity or impaired internal mobilization leads to early physiological collapse (Sperotto et al., 2014; Connorton et al., 2017). The superior performance of RA23 may arise from multiple tolerance mechanisms, including more efficient Fe(III)-chelate reduction, increased secretion of phytosiderophores, and improved root-to-shoot iron translocation—strategies widely reported in tolerant rice varieties (Ishimaru et al., 2006; Nozoye et al., 2019). The enhanced root architecture of RA23 likely further improves iron interception from the growth substrate, a feature underlying efficient nutrient foraging in iron-deficient soils. The PCA revealed interesting findings with the dominance of PC1 indicating strong correlations among several key traits, most likely due to underlying physiological linkages. PC1 was dominated by traits linked to root system size, leaf pigmentation and shoot biomass, it highlights that overall plant architectural robustness is the major source of multivariate variation among the genotypes studied. PC3 captured variation associated with shoot length and blade length, indicating a separate axis related to above ground traits. The presence of SPAD as the major contributor to PC8 further shows that chlorophyll content behaves independently of other traits and varies on its own axis. The consistent significance of Factor A in the ANOVA for all root traits shows that Fe concentration was the primary determinant of root system architecture in this experiment. The absence of significant genotype and interaction effects for root traits indicates that the influence of Fe levels on root development was stable and largely genotype-independent. In contrast, shoot traits such as leaf width and SPAD displayed significant genotype and interaction effects, showing that shoot physiology was more sensitive to genotype-specific responses under different Fe concentrations.

#### 4.1.1 SPAD as a trait for further transcriptomics analyses

SPAD was chosen because it captures a clear and biologically meaningful axis of variation, unlike many root and biomass traits that tend to move together. It has been used for years as a dependable indicator of leaf nitrogen status and photosynthetic capacity, and its measurements are known to be quite consistent (Peng et al., 1993; Monje and Bugbee, 1992). In the ANOVA, SPAD showed strong and significant effects for the main factors, along with a small but noticeable replication effect, which reflects both the strength of the signal and the precision of the measurements (Korte and Farlow, 2013). Since SPAD did not load with general growth traits on PC1, it clearly represents chlorophyll and nitrogen-related physiology rather than overall vigor. This independence is useful because the underlying molecular pathways are already well studied, making transcriptomic and GWAS results easier to interpret (Masuda and Fujita, 2008). Being non-destructive, quick and quantitative, SPAD also helps maintain clean phenotyping and leaves the tissue intact for RNA extraction, which is a clear practical advantage for both GWAS and RNA-seq work (Cortes et al., 2021).

### 4.2 Transcriptome quality and PCA confirm genotype-driven separation

The high-quality RNA-seq dataset generated in this study offers a robust foundation for dissecting iron-deficiency responses at the transcriptome level. Consistently high Q30 scores, strong read mapping rates, and uniform sequencing depth meet or exceed established criteria for high-confidence RNA-seq analysis (Conesa et al., 2016; Williams et al., 2019). These metrics confirm the technical reliability of the dataset and minimize the likelihood of artifacts in downstream analyses. Principal component analysis (PCA) further validated the biological coherence of the data, revealing strong genotype-driven separation between RA23 and LM. This pattern suggests that intrinsic genetic differences exert a dominant influence on basal transcriptional architecture, a phenomenon widely reported in nutrient-stress transcriptomics where tolerant and susceptible cultivars exhibit distinct regulatory states even prior to stress onset (Li et al., 2019; Yang et al., 2021). The tight clustering of biological replicates and the clear separation of iron-deficient samples from their corresponding controls indicate both experimental reproducibility and a strong treatment effect. Notably, the PCA patterns observed here mirror findings from previous work in rice, where iron-tolerant genotypes exhibit more stable or preadapted transcriptional profiles compared with susceptible ones, reflecting underlying regulatory differences that shape stress outcomes (Huang et al., 2019; Sharma et al., 2020). The alignment of genotype-specific clustering with physiological divergence strengthens confidence in the DEG patterns observed across contrasts and underscores that the observed transcriptional responses reflect true biological variation rather than technical noise. Taken together, the sequencing quality metrics and PCA outcomes confirm that the dataset is both technically sound and biologically meaningful. This provides a solid analytical framework for exploring the molecular mechanisms underlying iron-deficiency tolerance and susceptibility in the two rice genotypes.

### 4.3 Iron deficiency triggers distinct DEG architectures across contrasts

Iron deficiency elicited a highly structured and contrast-dependent transcriptional response in both genotypes, with each comparison revealing a unique DEG landscape shaped by developmental stage and stress intensity. The strongest transcriptional reprogramming was observed in the 0S vs 100S contrast, where hundreds of genes were differentially expressed. Many of these DEGs corresponded to hallmark components of the Strategy II iron-deficiency response in rice, including *YSL* transporters, *NRAMP* influx carriers, ferric-chelate reductases, and bHLH transcriptional regulators such as *IRO2* and *IRO3*. Similar large-scale induction of these genes has been reported in iron-starved rice and barley, reflecting the activation of phytosiderophore biosynthesis, metal chelation, and long-distance transport pathways (Kobayashi and Nishizawa, 2012; Aung et al., 2018; Nozoye et al., 2019). By contrast, the 0S vs 0T comparison revealed far fewer DEGs, dominated by stimulus and defense-associated processes rather than iron-specific signatures. Such non-iron, generalist regulatory patterns are widely documented in transcriptome studies of early developmental transitions and reflect baseline signaling shifts that precede nutrient-specific activation (Zhang et al., 2017; Chen et al., 2021). This indicates that iron-related transcriptional programs are not uniformly activated across early timepoints, and that biological context plays a key role in shaping DEG profiles. A similar pattern was seen in 0T vs 100T, where only a small set of up-regulated genes emerged. The limited magnitude of induction in this contrast resulted in insufficient statistical power for enrichment analysis, a phenomenon commonly observed when iron stress is mild, transient, or overshadowed by developmental cues (Ivanov et al., 2012; Kobayashi et al., 2019). These observations highlight that iron-deficiency responses are not strictly linear but depend heavily on temporal and physiological context. Genotype-dependent differences also contributed to the DEG architecture. In several contrasts, LM showed stronger transcriptional activation than RA23, consistent with the “over-activation” pattern often observed in susceptible genotypes that attempt to compensate for poor iron acquisition by hyperinducing transport and stress pathways (Sperotto et al., 2014; Ricachenevsky and Sperotto, 2014). In contrast, RA23 demonstrated a more selective and regulated expression shift—an adaptive strategy that has been associated with iron-efficient cultivars capable of minimizing reactive oxygen species accumulation and optimizing iron allocation (Briat et al., 2015; Huang et al., 2019). Such restrained yet effective transcriptomic modulation is considered a hallmark of physiological iron tolerance. Collectively, the DEG architecture across all comparisons illustrates a multilayered and condition-dependent response to iron deficiency. Strong activation of iron homeostasis genes under direct Fe limitation, contrasted with defense and stimulus centered profiles during early transitions, reveals the orchestrated interplay between developmental signals and nutrient sensing. These patterns underscore the complexity of iron-deficiency adaptation in rice and reinforce the fundamental differences between tolerant and susceptible genotypes.

### 4.4 GO enrichment highlights metal-handling and general stress pathways

GO enrichment analysis revealed clear contrast-specific biological themes. In 0S_vs_100S, iron deficiency triggered strong enrichment of *iron ion transport*, *transition metal transport*, and *intracellular metal homeostasis*, reflecting activation of the core iron-acquisition machinery described in previous rice and barley studies (Kobayashi and Nishizawa, 2012; Aung et al., 2018; Nozoye et al., 2019). The presence of oxidoreductase-related terms further supports the well-known link between iron deficiency and redox regulation (Briat et al., 2015; Bashir et al., 2013). In contrast, 0S vs 0T was dominated by *defense response*, *biotic stimulus response*, and *stress signaling* categories, consistent with literature showing that early developmental transitions frequently activate general stress pathways rather than metal-specific processes (Zhang et al., 2017; Chen et al., 2021). A similar pattern emerged in 0T vs 100T, where limited transcript induction resulted in enrichment only when UR and DR were combined, again yielding broad stress-associated terms with few iron-specific categories (Ivanov et al., 2012; Kobayashi et al., 2019). These patterns indicate that iron deficiency produces a dual-layered transcriptional response: strong activation of metal-handling pathways when iron deprivation is direct and substantial, and generalized stress-response signatures when developmental or mild-stress conditions dominate. Because the 0s_100s DEG set is small, ORA on separated UR and DR lists has reduced sensitivity; combining directions recovered additional metal-related terms. For a complementary, threshold-free view one could also apply a ranked GSEA on the full expression ranking to detect coordinated but small-effect shifts in metal-related pathways. Such context-dependent activation is well documented in rice and aligns with established models of iron-responsive regulatory networks (Ricachenevsky and Sperotto, 2014; Wang et al., 2020).

### 4.5 Distinct transcriptional strategies differentiate RA23 and LM

Clear genotype-dependent differences emerged in the magnitude and composition of transcriptomic responses to iron deficiency. In multiple contrasts, LM exhibited stronger induction of iron-responsive genes, including transporters and regulatory factors, whereas RA23 showed a more targeted and controlled transcriptional adjustment. This pattern of hyperinduction in susceptible genotypes has been documented in several crops, where plants with poor iron acquisition often compensate by excessively upregulating transport, chelation, and stress-related genes (Sperotto et al., 2014; Ricachenevsky and Sperotto, 2014). Such overactivation is frequently associated with inefficient iron utilization, higher oxidative burden, and impaired distribution of residual iron reserves (Connorton et al., 2017; Briat et al., 2015). In contrast, tolerant genotypes such as RA23 typically display restrained but efficient activation of iron-acquisition pathways. This regulatory strategy minimizes unnecessary energy expenditure and prevents the accumulation of reactive oxygen species, a known risk under iron-deprivation due to impaired electron transport and enhanced redox instability (Bashir et al., 2013; Huang et al., 2019). The selective transcriptional shifts observed in RA23—including modulation of transport, signaling, and redox-related components—align with the broader model that iron-tolerant cultivars rely on preconditioned or more fine-tuned transcriptional networks to maintain internal iron balance (Kobayashi and Nishizawa, 2012; Wang et al., 2020). Together, these genotype-specific transcriptional differences mirror the contrasting phenotypes observed. The hyperresponsive behavior of LM reflects a compensatory but inefficient strategy, whereas the moderated and coordinated transcriptomic adjustments in RA23 likely contribute to its superior maintenance of chlorophyll, root structure, and growth under iron deficiency. These findings reinforce the idea that transcriptional economy and regulatory precision are key determinants of iron-deficiency tolerance in rice.

### 4.6 Multi-parameter prioritization identifies robust candidate genes

The integrative prioritization strategy employed in this study allowed the identification of high-confidence candidate genes that may contribute to iron-deficiency responses beyond the well-characterized transporters and transcriptional regulators. By combining volcano plot outlier selection, curated annotation from RAP-DB, STRING-based interaction analysis, and cross-species homology searches, we were able to distinguish known iron-associated loci from poorly annotated or uncharacterized genes with strong computational evidence. This multi-layered approach reflects recent best practices in plant functional genomics, where integrating network context, evolutionary conservation, and domain prediction enhances the discovery of novel stress-responsive regulators (Wan and Liu, 2008; Usadel et al., 2009). Interestingly, several candidates lacked prior functional annotation yet displayed strong interaction connectivity to canonical iron-related pathways or consistent induction under deficiency, suggesting their potential involvement in iron homeostasis. Previous work has shown that uncharacterized or DUF-class proteins often occupy regulatory or structural roles in nutrient signaling and metal transport networks, despite their limited annotation (Finn et al., 2016; Goodstein et al., 2012). STRING-based associations further revealed that some of these candidates cluster near key iron regulators such as IRO3, YSL transporters, and NRAMP metal influx channels—patterns that have been used successfully in other studies to infer pathway membership and prioritize genes for validation (Snowdon et al., 2020; Pires and Dolan, 2012). The inclusion of transmembrane topology predictions (TMHMM) and PSI-BLAST–based homology searches strengthened the functional inference by distinguishing soluble proteins from membrane-associated candidates and by identifying lineage-wide conservation patterns. Such structural and evolutionary filters have proven effective in identifying membrane transporters, plastidial regulators, and stress-associated domains in rice and Arabidopsis (Sun et al., 2021; Guo et al., 2014). Together, this integrative screening pipeline allowed us to shortlist biologically plausible candidate genes, setting the stage for more targeted characterization. A particularly notable outcome of this filtering framework was the identification of OsFE97 as a strong candidate for further analysis, given its consistent induction under iron deficiency, conserved transmembrane architecture, and placement within a metal-responsive protein interaction neighborhood.

### 4.7 OsFE97 emerges as a novel plastid-associated iron-responsive gene

OsFE97 emerged as one of the most compelling previously uncharacterized candidates in this study, supported by its consistent transcriptional induction under iron deficiency, strong evolutionary conservation, and network-level association with established iron regulatory components. Although originally annotated as a DUF1230 protein with no assigned function, iterative PSI-BLAST searches revealed that OsFE97 belongs to the CGLD27-like family—an evolutionarily conserved group of plant and algal proteins with predicted plastid association. Orthologs such as At5g67370 in *Arabidopsis thaliana* and ycf36-like proteins in green algae have been implicated in stress-associated plastidial functions, suggesting that this family participates in conserved plastid signaling or metal-related processes (Eugenio et al., 2012; Friso et al., 2010). TMHMM-based topology predictions revealed a conserved architecture of three transmembrane helices across rice, *Arabidopsis*, and cyanobacterial homologs. This conserved transmembrane signature strongly supports a membrane-embedded plastid-associated role, consistent with plastid envelope proteins that modulate redox balance, metabolite exchange, or plastid-to-nucleus communication during nutrient stress (Pogson et al., 2015; de Souza et al., 2014). The identification of similar topology in distantly related homologs further underscores the evolutionary stability of this protein family and suggests a conserved biochemical role. Network-based evidence adds additional support for the functional relevance of OsFE97. STRING analysis placed this gene adjacent to major iron regulators including IRO3, a central bHLH transcription factor involved in orchestrating iron deficiency responses, and indirectly connected it to IRO2, YSL2, and NRAMP1, all well-characterized components of iron uptake and transport in rice (Kobayashi and Nishizawa, 2012; Aung et al., 2018). Although these predicted associations do not represent verified physical interactions, their clustering within a coherent iron-responsive module suggests that OsFE97 may act in coordination with, or upstream of, known metal-handling pathways. Such network-driven inference has been widely used to uncover new regulators within metal homeostasis networks in plants (Pires and Dolan, 2012; Guo et al., 2014). Importantly, OsFE97 displayed consistent upregulation in both RA23 and LM, with greater induction in LM—mirroring the broader hyperresponsive transcriptional behavior of the susceptible genotype. This pattern, combined with strong support from RT-PCR validation, indicates that the gene responds robustly to iron deprivation irrespective of genetic background. Given the predicted plastid localization and membrane topology, OsFE97 may participate in plastid-mediated regulation of iron distribution, redox homeostasis, or retrograde signaling, all of which are critical during iron limitation (Briat et al., 2015; Hantzis et al., 2018). Taken together, the structural, evolutionary, and network-level evidence strongly suggests that OsFE97 represents a previously unrecognized component of the rice iron-deficiency response. Its high conservation, plastid-associated features, and placement within an iron-responsive regulatory context make it a promising target for future functional dissection through localization assays, mutant analysis, and overexpression studies.

### 4.8 qPCR validation confirms reliability of RNA-seq expression patterns

The consistency between RNA-seq expression profiles and qPCR validation provides strong support for the reliability of the transcriptomic data generated in this study. Key iron-responsive genes, including established regulators such as IRO2, IRO3, YSL2, and NRAMP1, showed expression trends in qPCR that closely mirrored their RNA-seq-derived fold-changes, reaffirming the transcriptional activation of canonical iron-acquisition pathways under deficiency. Such agreement between sequencing-based and targeted validation approaches is widely regarded as a critical benchmark for confirming biological accuracy in transcriptomic studies (Bustin et al., 2009; Fleige and Pfaffl, 2006). Importantly, qPCR also substantiated the robust induction of the uncharacterized candidate OsFE97, supporting the computational inference that this gene is genuinely iron responsive rather than an artifact of sequencing or statistical filtering. The stronger induction observed in LM compared with RA23 aligns with the broader hyperresponsive transcriptomic behavior characteristic of susceptible genotypes, as reported previously for iron-inefficient rice cultivars (Sperotto et al., 2014; Ricachenevsky and Sperotto, 2014). This correspondence reinforces the biological relevance of genotype-specific transcriptional patterns identified here. Overall, the qPCR validation not only confirms the accuracy of the RNA-seq dataset but also strengthens confidence in the molecular trends identified, including those related to iron transport, regulatory signaling, and the newly characterized candidate gene OsFE97. The strong concordance between platforms provides a robust foundation for interpreting the gene networks and regulatory mechanisms underlying iron-deficiency tolerance in rice.

## 5 Conclusion

This study integrates physiological, transcriptomic, and computational evidence to clarify how two contrasting rice genotypes adapt to iron deficiency. RA23 maintained higher chlorophyll levels, growth, and root vigor than LM, reflecting more efficient iron homeostasis. Clear genotype separation in RNA-seq profiles and direct deficiency contrasts showed that RA23 activates metal transport, and plastid-associated pathways more effectively, while LM relies largely on general stress signaling. A major outcome is the identification of OsFE97 in rice which is an evolutionarily conserved CGLD27-like gene with plastid-localized transmembrane features (earlier reported in algae). Its consistent induction under iron deficiency, conserved structure, and association with core iron-responsive regulators (IRO3, IRO2, YSL2, NRAMP1) indicate a potential role in plastid metal handling or retrograde signaling. Validation through qPCR further supports its functional relevance. Overall, this work highlights the regulatory and transport strategies that underlie iron deficiency tolerance in rice and provides strong candidates, particularly OsFE97, for future functional studies. These insights offer promising avenues for breeding and biotechnological approaches to enhance iron-use efficiency and stress resilience in rice cultivated on iron-limited soils.

## Data Availability

The original contributions presented in the study are publicly available. The data presented in this study are available in the NCBS CAPS repository and can be downloaded at the following link: https://caps.ncbs.res.in/download/Iron_def_RNA_Seq_data/ All data are available from NCBI, accession PRJNA1431332. Supplementary data include primers used for the qRT-PCR analysis, functional annotation and gene ontology including functional domain and structure prediction analyses and DEG summaries, and are published alongside this article.
