# Supplementary material for: Comparative transcriptomic insights into iron deficiency response in contrasting rice varieties at the seedling stage reveal distinct response strategies and identify novel candidate genes
Source: Front Plant Sci. 2026 Mar 12;17:1764898. doi: 10.3389/fpls.2026.1764898 (PMC13019317; doi:10.3389/fpls.2026.1764898)
Supplement: Supplementary file 1 [file DataSheet1.pdf]

| Supplementary table 1 : Modified Yoshida's nutrient solution | | | |
|---|---|---|---|
| **Sl. No.** | **Chemical** | **Molecular weight** | **Yoshida's modified Solution ( gL-1)** |
| 1 | $NH_4NO_3$ | 80.04 | 91.40 |
| 2 | $K_2SO_4$ | 174.26 | 71.40 |
| 3 | $KH_2PO_4$ | 136.10 | 23.10 |
| 4 | $K_2HPO_4$ | 174.18 | 4.30 |
| 5 | $CaCl_2.2H_2O$ | 147.02 | 117.00 |
| 6 | $MgSO_4.7H_2O$ | 246.47 | 324.00 |
| 7 | $MnCl_2.4H_2O$ | 197.90 | 1.50 |
| 8 | $(NH_4)_6.Mo_7O_{24}.4H_2O$ | 1235.90 | 0.07 |
| 9 | $H_3BO_3$ | 61.83 | 0.93 |
| 10 | $ZnSO_4.7H_2O$ | 287.50 | 0.04 |
| 11 | $CuSO_4.5H_2O$ | 249.68 | 0.03 |
| 12 | FeNaEDTA | 367.00 | 0 (iron deficient) |
| 13 | $Ca(NO_3)_2$ | 164.08 | 207.13 |

| Supplementary table 2 : pH maintenance of the intended hydroponic environments across sampling days (5–18 DAS) | | | | |
|---|---|---|---|---|
| Day | 0% | | 100% | |
| | RA 23 | LM | RA 23 | LM |
| **5th** | 5.85 | 6.13 | 4.8 | 4.8 |
| **7th** | 5.36 | 5.55 | 4.36 | 4.39 |
| **9th** | 5.34 | 5.15 | 4.22 | 4.2 |
| **12th** | 6 | 6.15 | 4.45 | 4.2 |
| **15th** | 5.96 | 5.03 | 4.53 | 3.91 |
| **18th** | 5.83 | 5.88 | 4.34 | 4.03 |

| Supplementary table :3 - For every sample, 95.5–97.9% of reads passed filters, with GC content tightly clustered around 48.7–50.9% and duplication rates between 31.6% and 53.6% | | | | |
|---|---|---|---|---|
| **Sample name** | **% duplication** | **%GC content** | **% Passing filter** | **% Adapter trimmed** |
| LMAS-S-0per-I | 49.9 | 49.3 | 96.5 | 42.1 |
| LMAS-S-0per-II | 41.6 | 49.1 | 96.7 | 36.1 |
| LMAS-S-0per-III | 38.6 | 49.3 | 97.0 | 45.1 |
| LMAS-S-100per-II | 39.1 | 49.3 | 96.3 | 48.9 |
| LMAS-S-100per-III | 40.8 | 49.9 | 96.4 | 46.5 |
| RA23-S-0per-I | 53.6 | 48.7 | 96.7 | 38.4 |
| RA23-S-0per-II | 39.8 | 49.5 | 96.1 | 46.2 |
| RA23-S-0per-III | 31.6 | 48.8 | 95.5 | 47.4 |
| RA23-S-100per-I | 41.8 | 49.3 | 96.8 | 48.9 |
| RA23-S-100per-II | 36.4 | 50.9 | 97.7 | 28.9 |
| RA23-S-100per-III | 33.2 | 50.7 | 97.9 | 25.3 |
| LMAS-S-100per-I | 44.8 | 49.3 | 96.5 | 50.7 |

| Supplementary table 4: Comparative Expression Profiling of Commonly Upregulated (Fighter) Genes | | |
|---|---|---|
| **Gene ID** | **Fold Change (RNA-Seq)** | **Fold Change (RT-PCR)** |
| OsFE72 | 14.18011802 | 132.2303028 |
| OsFE28 | 10.82732672 | 63.01014174 |
| Os0FE99 | 6.675850475 | 45.06373184 |
| OsFE84 | 5.413504274 | 61.11565794 |
| OsFE93 | 3.558186054 | 53.11809677 |
| OsFE86 | 6.852137112 | 50.32982594 |
| OsFE20 | 7.437815825 | 87.15981046 |
| OsFE97 | 2.746477562 | 20.81441522 |

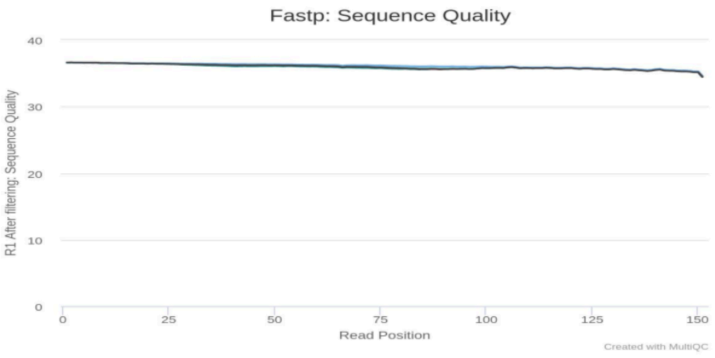


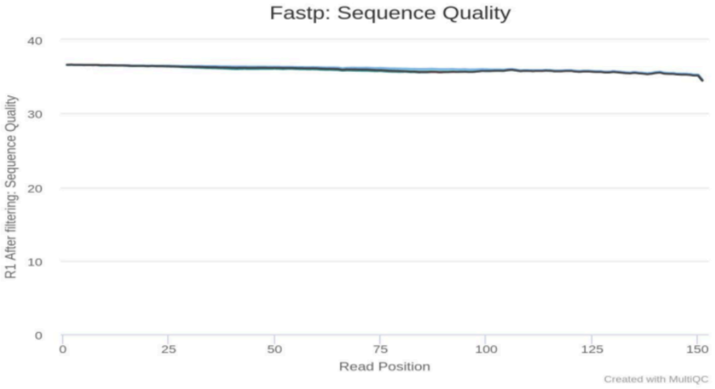


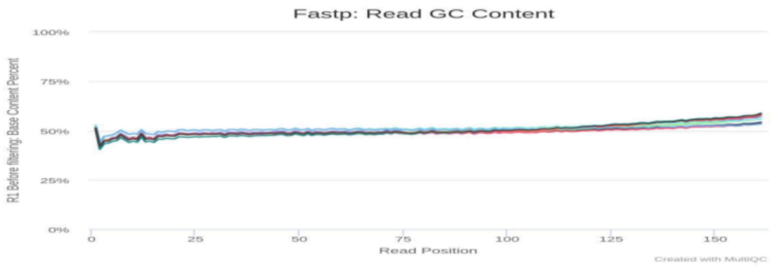


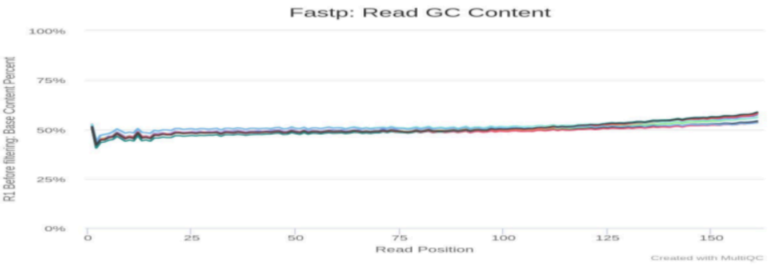


**Supplementary figure 1 : Per-base quality plots for Read1 and Read2**
